# Impact of ocean warming on sustainable fisheries management informs the Ecosystem Approach to Fisheries

**DOI:** 10.1038/s41598-017-13220-7

**Published:** 2017-10-18

**Authors:** N. Serpetti, A. R. Baudron, M. T. Burrows, B. L. Payne, P. Helaouët, P. G. Fernandes, J. J. Heymans

**Affiliations:** 1 Scottish Association for Marine Science, Scottish Marine Institute, Oban, PA37 1QA UK; 2 School of Biological Sciences, University of Aberdeen, Aberdeen, AB24 2TZ UK; 3 Sir Alister Hardy Foundation for Ocean Science, The Laboratory, Citadel Hill, Plymouth, UK

## Abstract

An integrated ecosystem model including fishing and the impact of rising temperatures, relative to species’ thermal ranges, was used to assess the cumulative effect of future climate change and sustainable levels of fishing pressure on selected target species. Historically, important stocks of cod and whiting showed declining trends caused by high fisheries exploitation and strong top-down control by their main predators (grey seals and saithe). In a no-change climate scenario these stocks recovered under sustainable management scenarios due to the cumulative effect of reduced fishing and predation mortalities cascading through the food-web. However, rising temperature jeopardised boreal stenothermal species: causing severe declines in grey seals, cod, herring and haddock, while eurythermal species were not affected. The positive effect of a higher optimum temperature for whiting, in parallel with declines of its predators such as seals and cod, resulted in a strong increase for this stock under rising temperature scenarios, indicating a possible change in the contribution of stocks to the overall catch by the end of the century. These results highlight the importance of including environmental change in the ecosystem approach to achieve sustainable fisheries management.

## Introduction

Overexploitation of natural resources is one of the greatest anthropogenic pressures impacting the structure and functioning of marine ecosystems over short time scales^1,2^. Changes in fish communities showing shifts in the trophic-web and declines in mean trophic level have been observed in Europe^3–6^. The ecosystem approach to fisheries (EAF) aims to achieve sustainable fisheries by combining broad ecological sustainability of stocks with the socio-economic viability of the fishing industry at local and regional scales^7^. This approach is design to ensure a sustainable state of marine food webs (i.e. no overexploitation), allowing continued fishing to support human well-being. While current fisheries management relies mostly on single species assessment models, recent studies have underlined the importance of implementing EAF to explore fishing management scenarios^7–9^. However, EAF requires ecosystem models to be parameterised over a historical time period to enable forecasts of future fish biomass.

Fishing-induced ecosystem changes often coincide with rising temperatures driven by climate change, requiring climate-change effects to also be considered in model forecasts. An integrated methodology including the temperature tolerances of species is therefore needed to assess the impact of climate change on fisheries^10^, on ecosystem diversity^11^, and the social-ecological responses to potential ecosystem changes^12^.

Both climate variability and climate change affect marine ecosystems: ‘climate variability’ is a natural short-term fluctuation over a long-term average^13^ such as ocean-atmosphere coupling phenomena and decadal oscillations. Climate variability has often been identified as a major driver of ecosystem dynamics^14^, and quantified using indicators such as the Pacific Decadal Oscillation (PDO)^15^, El Niño-Southern Oscillation (ENSO)^16,17^, Atlantic Multidecadal Oscillation (AMO)^18–21^ and North Atlantic Oscillation (NAO)^22^. Climate change, on the other hand, refers to the long-term anthropogenic-mediated modifications of the Earth’s climate^13^ caused by global increases in gas emissions and its subsequent consequences (e.g. rising temperatures). Global-scale climate models showed a rapid increase of ocean temperature in the last forty years with a global average increase of 0.11 °C per decade^23^. Climate change has been recognised as one of the greatest threats to biodiversity of this century^11^, affecting the integrity of ecosystem resources^24–28^.

Although oceans exhibit a slower warming trend than land, the distributions of pelagic, demersal and benthic marine species have shown higher rates of poleward migrations than terrestrial species due to fewer physical barriers in the oceans^29^. However, even in the marine environment, physical barriers (e.g. current, gyre, trenches), lack of suitable habitat (e.g. topography, depth, oxygen) and antagonistic trophic interactions (e.g. competition and predation) can represent barriers to temperature-driven poleward dispersal^30^. These barriers to dispersal make some species more vulnerable to climate change than others^24,31^. Poleward distribution shifts are increasing the relative presence and abundance of warm-water species in mid- to high-latitude regions (such as the Bering Sea, Barents Sea, Nordic Sea, North Sea, and Tasman Sea) and thus affecting community functioning and diversity^32,33^.

Predicting the impact of climate change is challenging given the specific responses of marine organisms at regional scales, and the cascading effects of these responses (synergistic or antagonistic) on the entire ecosystem^34–36^. Anthropogenic pressures, such as fishing, pollution, eutrophication and habitat modification, are increasing ecosystem vulnerability by decreasing resilience, adding even more complexity to the process of assessing the impact of climate change at a local scale^36^. Species populations and distributions are also regulated by competition, predation and environmental and human pressures. Ecosystem modelling approaches address these complexities directly and can help to understand and predict ecosystem shifts. Despite being more realistic, however, predictions from complex models that include ecological interactions usually have a high degree of uncertainty^37^.

Reducing uncertainty in predictions of climate-induced changes in ecosystems is therefore needed to understand their societal consequences. A better understanding of the nature and scale of the response of marine species to climate change will improve predictions of the ecological and economic impacts on human systems, and contribute towards management mitigation strategies^29,38^. In cold water ecosystems, for example, local native species might be negatively affected by higher temperatures, but the increase in the abundance of warm water species may allow exploitation of new stocks^39^.

In this study, we used an Ecopath with Ecosim (EwE) ecosystem model of the West Coast of Scotland (WCS)^40^ to assess the combined impacts of fishing and rising temperature on species consumption. The model (which included 41 functional groups^40^) was parameterised with species thermal tolerances and used to simulate the impact of fishing by comparing status-quo and maximum sustainable yield (MSY) scenarios, and to assess the impact of climate change on important commercial stocks. The impact of climate change was tested using future rising temperature under IPCC Representative Concentration Pathways (RCPs) scenarios, while keeping fishing pressure constant at rates deemed consistent with single species MSY. Results were presented for target species identified as either depleted stocks (Atlantic cod, whiting and herring), increasing stocks (saithe and hake), and other important contributors in terms of landings (haddock, mackerel, horse mackerel and Norway lobster), as well as certain top-predators (grey seals) that exert top-down control on the ecosystem^41,42^.

## Results

### The model

The initial model (Supplementary Tables S1–S6), without temperature as an ecosystem driver, showed a relatively high sum of squares (SS) between predicted and observed data for the baseline (1620) and also when including fishing (1219) (Table 1).

**Table 1:** Comparison across selected stepwise fitting interactions and the model baseline, showing the number of total parameters estimated (Vulnerabilities (Vs) + number of anomaly spline points (PP_anomaly)), the model sum of squares (SS), the SS percentage of contribution to the fitting, the Akaike Information Criterion (AIC) and the AIC weights.

	Name	Parameters estimated	SS	contribution to SS fitting	AIC	Akaike weight
without temperature	Baseline	0	1620	—	256	0
	Fishing	0	1219	25%	−151	0
	**Fishing + 24 Vs + 3 PP_anomaly**	**27**	**614**	**62%**	−**1079**	**0.61**
with temperature	Baseline	0	1515	—	158	0
	Baseline + 33 Vs	33	1033	32%	−323	0
	Baseline + 5 PP_anomaly	5	1484	2%	139	0
	Fishing	0	1126	26%	−266	0
	Fishing + 33 Vs	33	793	48%	−702	0
	Fishing + 5 PP_anomaly	5	1038	32%	−372	0
	Fishing + 24 Vs + 3 PP_anomaly	27	632	58%	−1040	0
	**Fishing + 33 Vs + 5 PP_anomaly**	**38**	**607**	**60**%	−**1076**	**0.25**
	Fishing + 36 v + 3pp	39	606	60%	−1075	0.15
	Fishing + 36 v + 4pp	40	603	60%	−1073	0.06

In bold the best fitted models.

The model was fitted to observed time-series identifying the best fitted model by the lowest Akaike Information Criterion (AIC) (Table 1). Adding fishing, the strength of trophic interactions expressed as vulnerabilities (Vs), and the impact of a primary productivity anomaly (PP_anomaly) applied to ecosystem primary producers, increased the fit of the model reducing the SS by 62% and reducing the AIC from 256 to -1079 (Table 1). The Akaike weight for this interaction also indicated a 0.61 probability that this is the best fitted model when not including temperature (Table 1).

**Figure 1:**
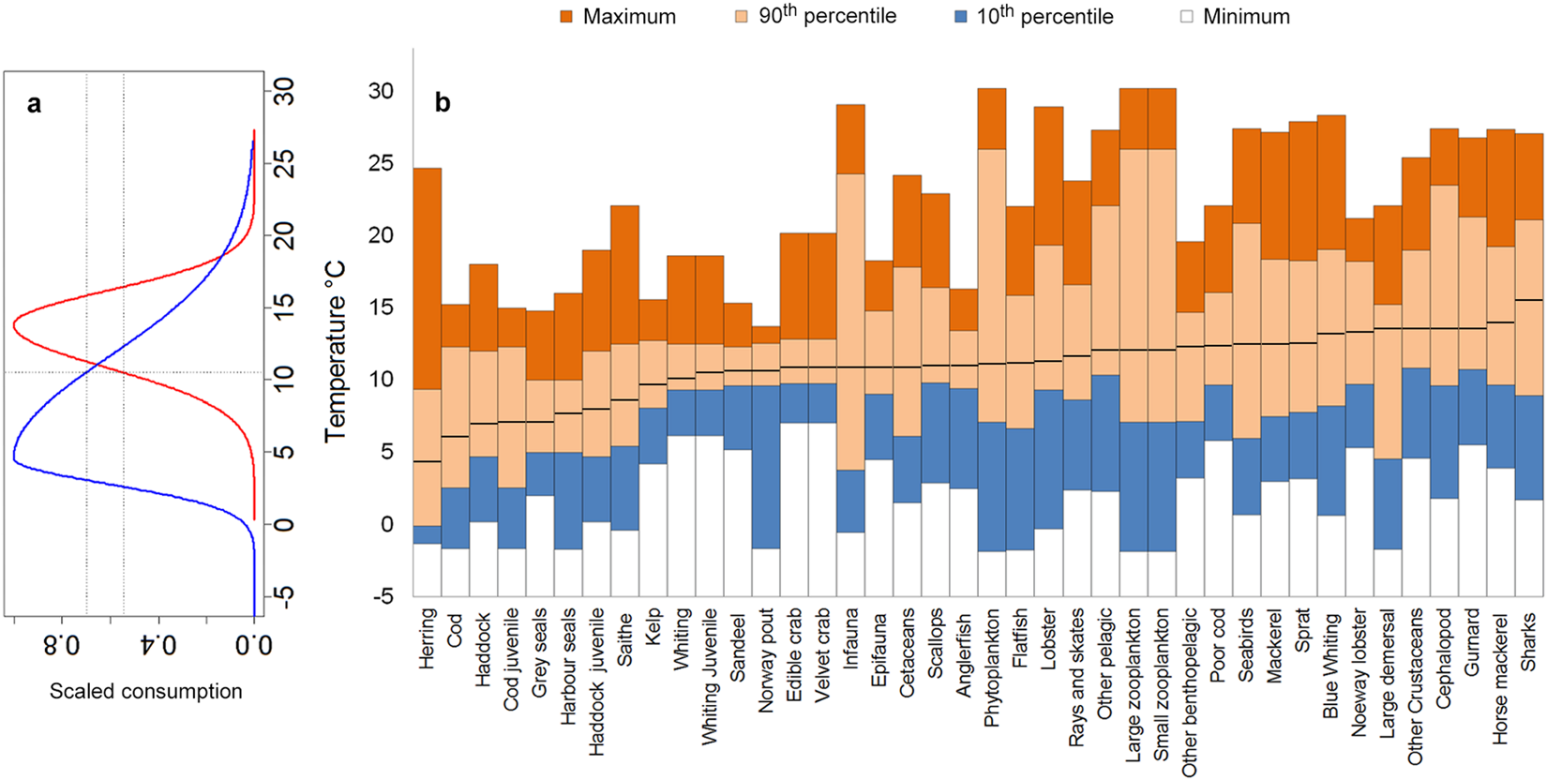
(**a**) Species thermal response functions (for Norway lobster, a eurythermal species (red, optimum temperature = 13.8 °C) and for herring, a boreal species (blue, optimum temperature = 4.6 °C)). The intercept between water temperature (e.g. 10.5 °C) and the species response functions determined the consumption rate scaling factor (i.e. 0.54 and 0.7 for Norway lobster and herring respectively). (**b**) Cumulative temperature tolerance graphs ranked by optimum temperature (bold black line) also showing maximum (upper limit of dark orange bar) and minimum (upper limit of white bar) temperatures and the 90^th^ (upper limit of light orange bar) and 10^th^ (upper limit of blue bar) percentiles for each functional group.

Optimum temperatures and thermal tolerances of each functional group were then added into the model as response functions to water temperature (Fig. 1a). The species temperature tolerances ranked by optimum values (Fig. 1b) show the preference of lower optimum temperatures and tolerances for north Atlantic boreal species such as herring (blue, Fig. 1a), and Atlantic cod, haddock, grey and harbour seals, saithe and kelp, and higher optimum temperatures and tolerances for more widely distributed eurythermal species such as horse mackerel, Norway lobster (red, Fig. 1a), blue whiting, sprat and mackerel. The tolerances around optimum temperatures for eurythermal species were heterogeneous across species often showing lower tolerances for benthic-demersal groups (epifauna, anglerfish, flatfish, other benthopelagic species, poor cod, and Norway lobster) (Fig. 1b).

**Figure 2:**
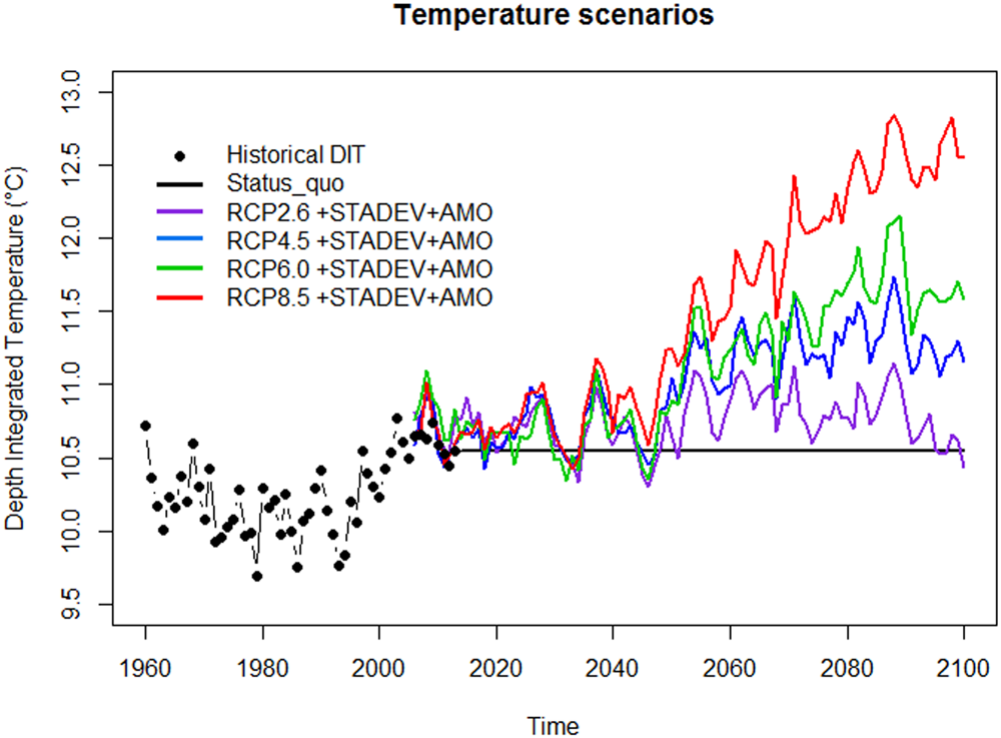
Depth integrated temperature (DIT) data used in Ecosim simulations. Historical DIT data (black points) were used to parameterise the model whilst status quo (constant water temperature measured in 2013 – black solid line) and modified climate changing multi-model ensemble means (RCP2.6–solid purple line; RCP4.5–solid blue line; RCP6.5–solid green line; RCP8.5–solid red line) were used for predicting future scenarios.

The addition of temperature response functions by functional group (Fig. 1a) (Supplementary Table S8) combined with depth integrated water temperature (DIT, black points in Fig. 2) reduced the AIC for the baseline (i.e. no fishing) to 158 from 256 and for the model with fishing interactions to -266 from -151 (Table 1). The addition of temperature as an ecosystem driver with the related functional group temperature niches also allowed the simulation of future DIT temperature scenarios (Fig. 2).

**Figure 3:**
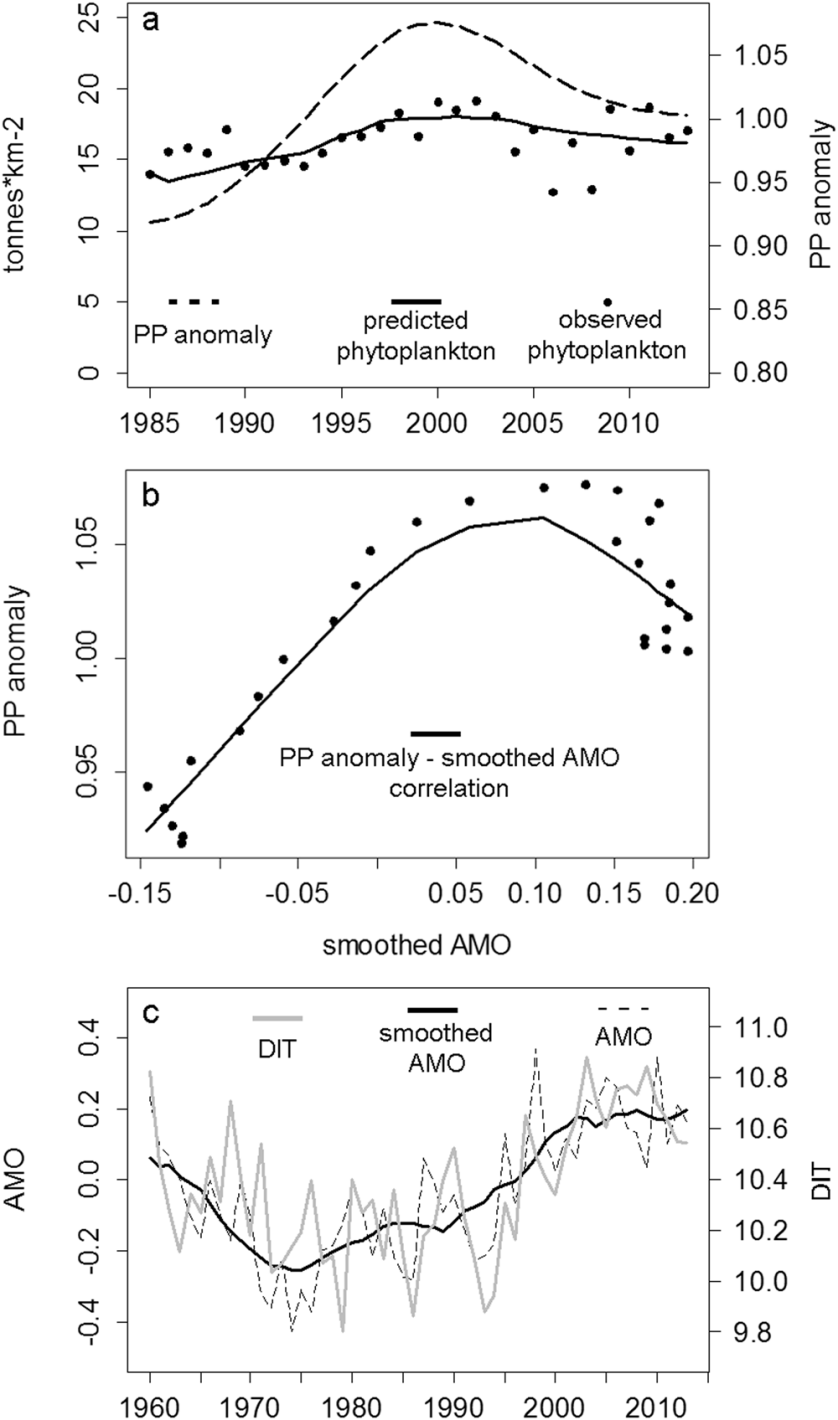
(**a**) Temporal trends of predicted (black line) and observed (points) phytoplankton biomass vs the primary production anomaly (PP_anomaly, dashed line) showing the potential effect of the PP_anomaly on improving the phytoplankton fitting; (**b**) Loess function showing correlation of PP_anomaly and smoothed AMO; (**c**) correlation between AMO and depth integrated temperature (DIT).

The best-fit model with temperature showed an improvement in fit as predicted by the SS of 60% reducing the AIC from 158 to -1076 (Table 1). It included fishing, 33 vulnerabilities (Vs, Supplementary Table S7) and a PP_anomaly function with 5 spline points (Fig. 3a). In comparison with the baseline model, the inclusion of the trophic interactions only (e.g. 33 vulnerabilities) improved the SS of the model fit by 32% (Table 1). Only including the anomaly (with 5 spline points) had the lowest effect (2%), reducing the AIC to 139 from 158 (Table 1). The Akaike weights indicated a range of models with an AIC of approximately -1076 which could be the best fitted model (Table 1).

Within the 33 estimated vulnerabilities, four top-predators showed sensitive top-down control on their prey (i.e. v*ij* > 2): grey seals, cetaceans, mature cod and saithe, whist pelagic fish (i.e. benthopelagic fish and other pelagic fish groups) as well as large and small zooplankton, and benthic groups such crabs, other benthic crustaceans, and scallops were all bottom-up controlled with 1 < v*ij* < 2 (Supplementary Table S7).

The PP_anomaly function estimated by the model hindcast from 1985-2013 is significantly correlated with the smoothed AMO climate function (Fig. 3b, Table 2). Both smoothed and unsmoothed AMO were significantly correlated with Sea Surface Temperature (SST) (Fig. 3c, Table 2). No correlation was found between the PP_anomaly and the NAOI or the SST (Table 2).

**Table 2:** Spearman correlation values and statistical significance within SST, AMO, NAOI and PP_anomaly.

R^2^, *p-value*	SST	AMO_smoothed	AMO_unsmoothed	NAOI	PP_anomaly
SST	1	**0.65**	**0.67**	0.36	0.50
AMO_smoothed	***	1	**082**	−0.22	**0.72**
AMO_unsmoothed	***	***	1		
NAOI	**	–	**	1	0.25
PP_anomaly	**	***	**	–	1

Symbols indicate the levels of significance: ***p < 0.001, **p < 0.01, *p < 0.05 and –no significance.

### Model validation and uncertainties of the predictions

Model validation analysis showed an overall progressive improvement described by decreasing root mean square deviations (RMSD) between predicted and observed biomass values when using more data in the model fitting process. Within the target functional groups, biomass predictions of large demersals, mackerel and saithe showed large deviations from observed data across all the validation subsets (A, B and C in Table 3 and Supplementary Fig. S1). Large deviations from observed catches were also predicted for mackerel, although no clear pattern of prediction deviations was found within the validation datasets (Table 3 and Supplementary Fig. S1).

**Table 3:** Root mean square deviations (RMSD) across three validation subsets (A, B and C) for biomasses and catches for the target and all functional group.

Functional group	Biomass			Catches		
	A (RMSD)	B (RMSD)	C (RMSD)	A (RMSD)	B (RMSD)	C (RMSD)
Grey seals	0.0040	0.0070				
Cod	0.0423	0.0151	0.0161	0.0286	0.0113	0.0114
Haddock	0.1516	0.1778	0.0776	0.0615	0.0168	0.0331
Whiting	0.2556	0.0564	0.0657	0.0412	0.0027	0.0024
Saithe	2.3383	2.7239	3.9048	0.0514	0.3162	0.5335
Large demersals	3.8231	2.0891	0.4547	0.1402	0.1188	0.1209
Mackerel	1.5819	1.2381	1.1768	0.4489	0.2841	0.2378
Horse mackerel	1.2457	1.0594	1.3777	0.3380	0.2806	0.4318
Herring	0.6954	0.5579	0.4296	0.1394	0.1700	0.0389
Norway lobster	0.2149	0.3054	0.3570	0.0494	0.0475	0.0474
Total model functional groups	1.9194	1.6402	1.2316	0.1461	0.3921	0.1690

Subset A: 22 years for model fitting, 7 years validation set; subset B: 24 years for model fitting, 5 years validation set; subset C: 26 years for model fitting, 3 years validation set).

In the Monte Carlo analysis, using both 10% fixed variability of the model inputs and the input data pedigree (Supplementary Table S4) showed higher uncertainties in the predictions of cod, haddock and whiting species as seen in the 95% and 5% percentiles (Supplementary Fig. S2).

### Predicting cumulative effects of rising temperature and fishing pressure on target species biomasses and catches

**Figure 4:**
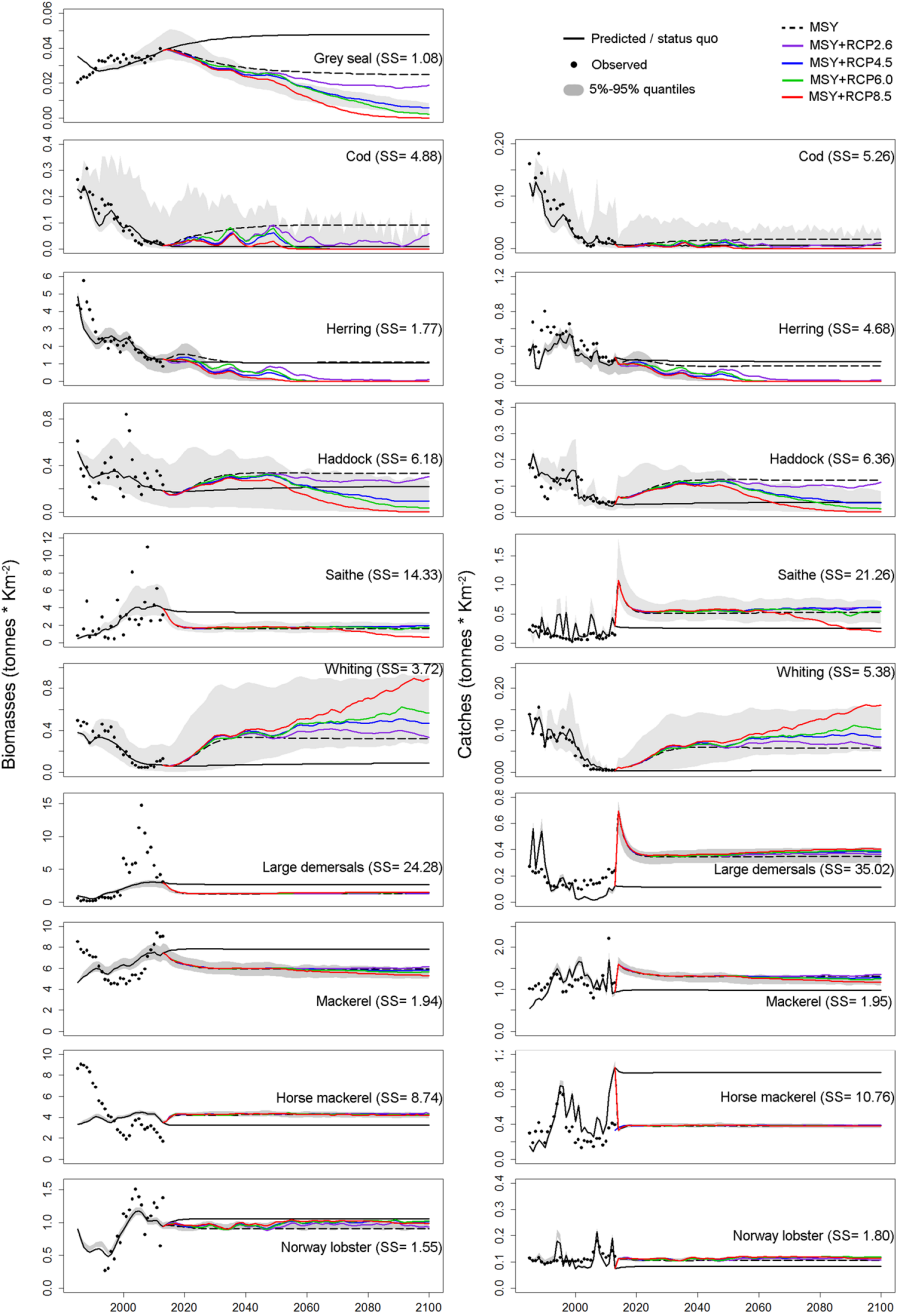
1985-2013: fitted observed (black data points) and hindcasted model output (solid black line) with 95% and 5% percentiles of the Monte Carlo simulations (shaded area). Monte Carlo simulations for future prediction are plotted only under the “MSY + RCP4.5” scenario sake of clarity. 2014–2100: future model predictions for “status quo” (solid black line), “MSY” (dashed black line) and for “IPCC-RCP” scenarios (MSY + RCP2.6–solid purple line, MSY + RCP4.5–solid blue line, MSY + RCP6.5–solid green line, and MSY + RCP8.5–solid red line) for species biomasses (left panels) and catches (right panels).

The hindcast model output (Fig. 4, solid black line 1985-2013) fitted the observed data trends (Fig. 4, black data points and Sum of Squares (SS) contributions) for most of the target species, such as grey seals, cod, haddock, whiting, saithe, large demersals, herring, and Norway lobster. From 2000 onwards the model produced a poor fit for the biomass and catch trends of large demersals. A poor fit was also evident for mackerel and horse mackerel biomasses, although a better fit was found for catches of these species. Contrasting results were obtained for saithe, with good estimates of the increasing trend of biomass, but very scattered catch predictions for this species. The model performed well in estimating trends of catches for all other target species. The 95% percentile of the Monte Carlo simulations with 10% variability of the model inputs (shaded areas in Fig. 4, and Supplementary Fig. S2) exhibited high variability for cod, due to water temperatures being at the edge of their temperature tolerance (Fig. 1a,b), hence their temporal consumption rates varied substantially with feeding time and vulnerability parameters (Eq. 1 in Methods).

Future changes of biomass and catches were assessed under constant and rising temperature scenarios (Fig. 2). Under the “status quo” future scenario (Fig. 4) grey seal biomass was predicted to follow the increasing historical trend, reaching equilibrium by 2050. However, under the “MSY” scenario their biomass decreased to values similar to the 1990s. This decrease is due to top-down competition with fishing fleets targeting the prey species of grey seals (saithe, cod, haddock, whiting and large demersal). With fishing mortality set at F_MSY_ (Table 4 in method section), the predicted increase in biomass of prey species, such as cod, haddock and whiting, did not compensate for the decrease in biomass of saithe and large demersals. The reduction in grey seals is thus driven by the reduction of their prey. In the “MSY + RCP2.6” scenario, grey seal biomass slightly decreased compared to the “MSY” scenario, but remained constant to the end of the century. Grey seal biomass decreased in all the other rising temperature scenarios. A total collapse of this species was predicted by 2090 for the worst case IPCC scenario (MSY + RCP8.5).

**Table 4:** Target species fishing mortality at the “status quo” (F_status_quo_) and at the maximum sustainable yields (F_MSY_).

Species	F_status_quo_	F_MSY_	Reference
Cod (*Gadus morhua*)	0.6	0.19	ICES^94^
Haddock (*Melanogrammus aeglefinus*)	0.17	0.37	ICES^94^
Whiting (*Merlangius merlangus*)	0.055	0.18	ICES^95^
Saithe (*Pollachius virens*)	0.07	0.32	ICES^94^
Mackerel (*Scomber scombrus*)	0.13	0.22	ICES^96^ (western shelf)
Horse mackerel (*Trachurus trachurus*)	0.3	0.13	ICES^96^ (western shelf)
Herring (*Clupea harengus*)	0.21	0.16	ICES^97^
Hake (*Merluccius merluccius*) (85% of large demersal)	0.04	0.27	ICES^94^ (northern stock)
Anglerfish (*Lophius piscatorius & L. budegassa*)	0.14	0.19	ICES^94^ (Bay of Biscay)
Blue Whiting (*Micromesistius poutassou*)	0.11	0.3	ICS^94^
Norway lobster (*Nephrops norvegicus*)	0.08	0.116	ICES^94^ (underwater TV surveys, average within FU 11, 12 and 13)

In the “MSY” scenario, the biomass of cod, haddock, whiting and herring increased due to (i) F_MSY_ being lower than F_status quo_ (Table 4 in methods section), (ii) lower predation rates from grey seals and saithe on juveniles, or (iii) a combination of both. The climate scenarios showed different responses to rising temperature across species. Cod and herring, with low optimum temperatures (Fig. 1), were sensitive to rising temperature and strongly declined by 2060 under all the climate projections. Cod also showed large oscillations due to temperatures being close to the upper end of their tolerance (Fig. 1a,b), causing large variations in their ability to feed over time (Eq. 1 in Methods). Under the “MSY + RCP2.6” scenario, cod biomass increased by the end of the century associated with the lower temperatures of this climate scenario (Fig. 4 and Fig. 2). Haddock biomass was more resilient to warming; maintaining constant biomass under the “MSY + RCP2.6” scenario, but a large decline in other RCP projections. Saithe’s optimum temperature is higher than that of cod and haddock, but lower than whiting. Saithe is also more eurythermal than all the other gadoids (Fig. 1b) and declines only under the “MSY + RCP8.5” scenario. Whiting biomass only remained stable under “MSY + RCP2.6” scenario, but showed a strong increase under the other RCP projections. These increasing trends were driven by the cumulative effects of higher optimum temperature for this species compared to the other gadoids (Fig. 1b) and low predation pressure due to the predicted declines of their main predators (grey seals and cod) (Fig. 4).

Eurythermal species and those in the cooler half of their thermal range (Fig. 1a), such as large demersals (consisting of 85% hake), mackerel, horse mackerel and Norway lobster, did not show any changes under rising temperature projections (Fig. 4). The effects of fishing are more evident for catch predictions, with large variations under the “MSY” scenario projected for saithe, large demersals, mackerel and horse mackerel, due to a sudden increase in F_MSY_ from the status quo (Fig. 4).

**Figure 5:**
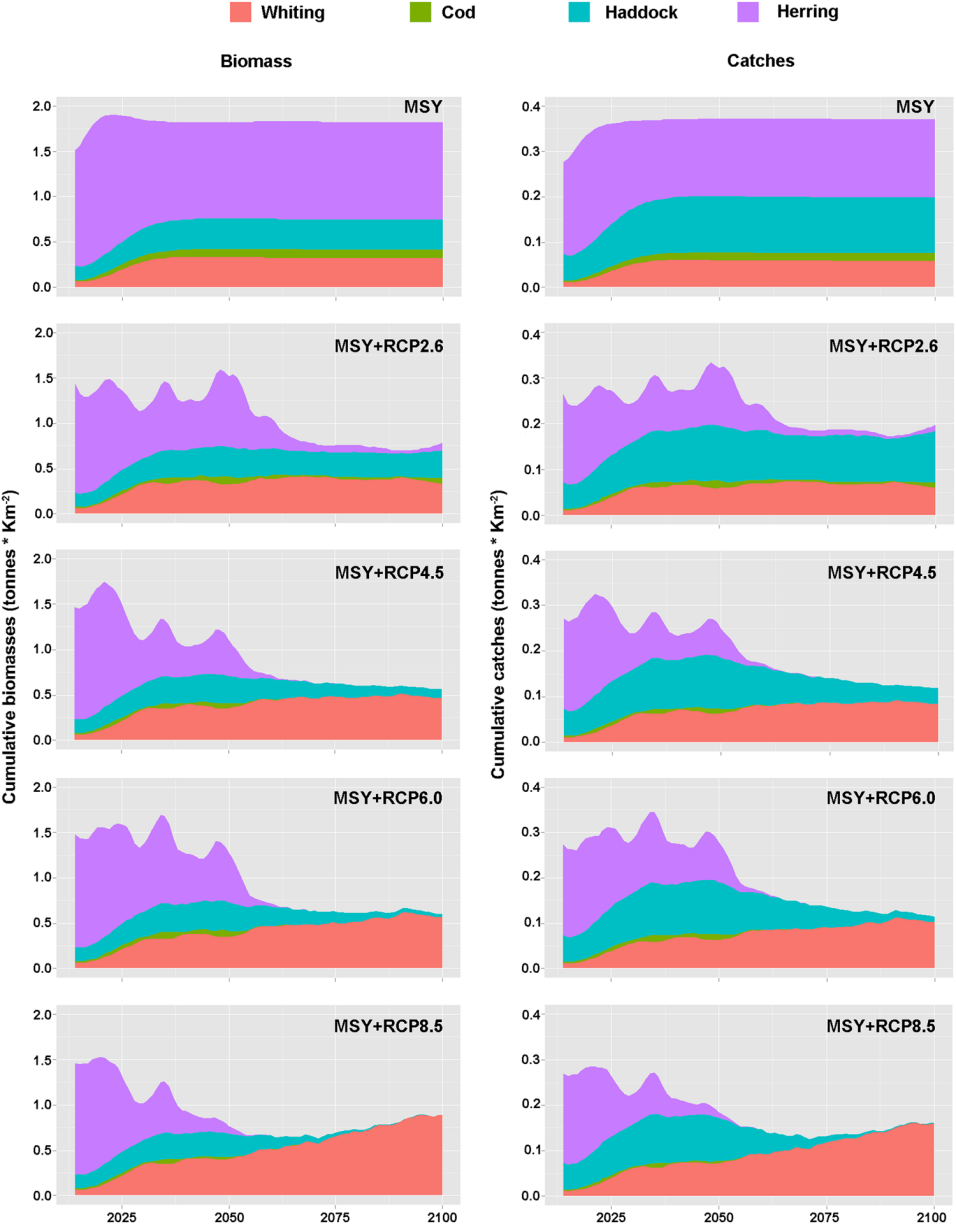
Cumulative biomasses (left panels) and catches (right panels) of whiting (orange), cod (green), haddock (light blue) and herring (purple) under MSY and IPCC-RCP scenarios.

Mackerel, horse mackerel and saithe were the dominant species in terms of biomasses and catches (Fig. 4). Under the highest rising temperature scenario (MSY + RCP8.5), overall decreases of 15% and 20% for cumulative biomasses and catches respectively were predicted for these three species by 2100 (from 15.8 to 13.5 tonnes *km^−2^ for cumulative biomasses and from 3.04 to 2.42 tonnes *km^−2^ for cumulative catches). Of the fished species, cod, herring, haddock and whiting showed major changes in response to rising temperatures (Fig. 4). The cumulative biomasses and catches for these species showed an overall decreasing under all the IPCC scenarios compared to the “MSY” scenario (Fig. 5). Under the highest temperature scenario (MSY + RCP8.5) the large increase of whiting stock predicted by 2100 starts to compensate for the losses of other target species (Fig. 5), although it does not quite make up for the reduction in herring and haddock.

## Discussion

Using an ecosystem modelling framework that accounts for environmental and human pressures alongside species ecology and biology, we built an intricate web of interactions with the aim of predicting future ecosystem changes to ensure sustainable exploitation in a changing environment. We used the WCS EwE model developed in 2005^43^ and recently refined^40,44^ to assess the cumulative synergistic or antagonistic effects of rising temperatures and fishing on the marine ecosystem.

Model validation (Table 3 and Supplementary Fig. S1) showed reduced fit on predicting biomasses of large demersals, saithe and mackerel due to large observed increases of both saithe and large demersals since 1985 (Fig. 4), and the migratory ecology of mackerel which is only in the study area for a quarter of the year^45^. An improvement in out-of-sample predictability was found for overall biomass predictions as the number of observations in the model calibration increased. This pattern was not observed for catches, however, probably due to the complex ecosystem dynamics that often show non-linear and non-stationary dynamics and require longer time series to be detected^46^.

Monte Carlo simulations assessing the prediction uncertainties with changes in the input data showed larger variabilities for cod, haddock and whiting compared to saithe, large demersals, Norway lobster and pelagic species such as mackerel, horse mackerel and herring. These variabilities for cod, haddock and whiting are caused by the top-down and bottom-up interactions between the adult and juvenile stages of these multi-stanza groups (Supplementary Table S6).

The primary production anomaly (PP_anomaly) predicted by our model showed similar trends ‘with and without’ temperature as a driver in the model, and were comparable with previous anomalies estimated by Alexander *et al*.^40^ for this ecosystem. The hindcast biomasses and catches for target species between the best models ‘with and without’ temperature also showed similar trends. The significant correlation found between the AMO and the PP_anomaly (Fig. 3b), confirmed that the anomaly represent a potential ecosystem driver^21,47^. Previous studies found opposite correlations between climate variability indices and primary productivity, indicating the complexity of their effects on different ecosystems^15,19^. Our results however reinforced the importance of the AMO as a potential climate driver in the North Atlantic ecosystem, supporting the positive temporal and spatial correlations between AMO and primary productivity^21^.

**Figure 6:**
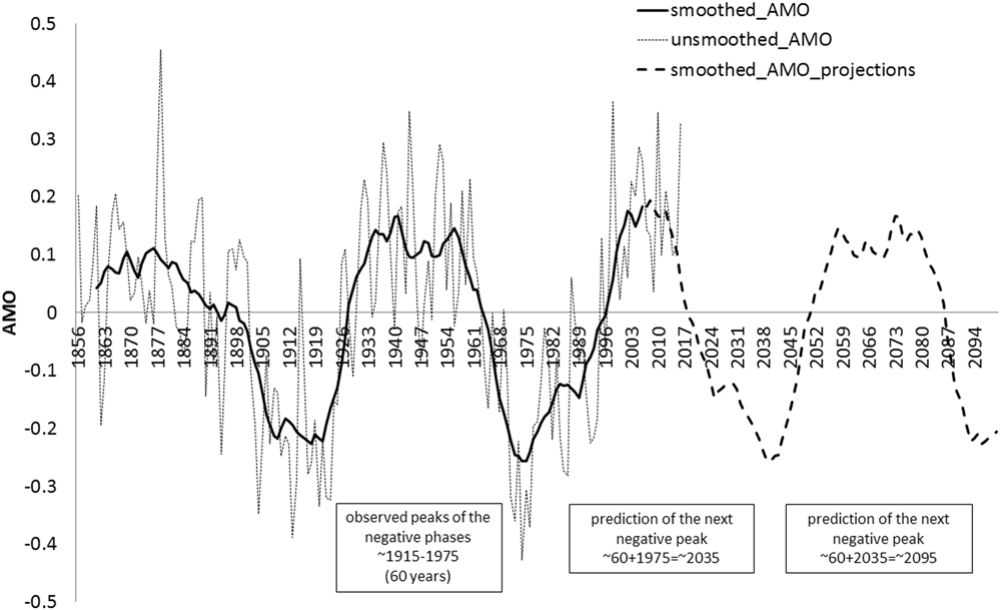
Historical unsmoothed (fine-dashed line) and smoothed (solid line) AMO data. Future projections (coarse-dashed line) were created mirroring AMO smoothed data from 2012 to 2100 to allow the re-occurrences of the lower peaks as for the historical data at intervals of approximately 60 years.

The AMO has a distinct spatial distribution with a strong signal in the North Atlantic^48,49^, underlining the necessity of integrating the AMO signal into the de-trended IPCC temperature rates extracted from the study area (Supplementary Fig. S4) for predicting the rising depth-integrated temperature (DIT) scenarios (Fig. 2 and method section). The AMO is now approaching the start of its ‘cooling’ phase (Fig. 6)^50^, potentially slowing down the rates of temperature rise for the next 30 years, and underlining the importance of implementing this index on future temperature predictions to mitigate the predicted declines of boreal stenothermal species.

The recent increase in the biomass of grey seals and saithe (observed data, Fig. 4) was well predicted by our model. These are key-species in regulating the equilibrium of the ecosystem by exerting top-down control on their prey (v*ij* > 2, Supplementary Table S7). These results echo recent studies which underlined the importance of grey seals in the WCS. Grey seals have been increasing in abundance since the 1960s mainly because of the increasing availability of their prey^51^ combined with few natural predators. According to our results they can play an important role in preventing the recovery of the overexploited cod stock, which also support previous findings^41,42^. The difference in grey seal biomass projections between the “status quo” and “MSY” scenarios indicated that good ecosystem level fisheries management could have an important impact on regulating the population of top-predators and reduce their top-down control on the ecosystem.

Low historical fishing and predation mortalities for saithe and large demersals caused large increases in biomass for both these species, whilst high predation and fishing rates caused large declines of cod and whiting (Fig. 4). Saithe increased fourfold between 1985 and 2013 due to low fishing and predator pressure. Conversely, the biomass of the large demersal group constituted 80-85% of hake, which showed a dramatic increase since the early 2000s in both the North Sea and the West of Scotland^52^. However, our parameterisation of large demersals also include the life history parameters, diet distribution, etc. of other species included in this functional group (Supplementary Table S8). This might explain the poor performance of our model in predicting trends in biomass and catches of large demersals, and underlines the necessity to consider hake individually for future applications of this model, especially given the large increase in this species biomass in recent years^52^. Under the “MSY” scenario, grey seals declined due to competition with fishing fleets that target their prey (mainly saithe and large demersals) whilst the drop in numbers of saithe and large demersals were caused by a higher fishing mortality (F_MSY_ > F_status_quo_): the cumulative effect of the reduction in top-predators determined the improvements of the yields of the cod and whiting declining stocks. This prediction could not be identified and predicted by single species assessments, emphasising the importance of multi-species considerations in an ecosystem approach to fishery management.

“IPCC-RCP” scenarios were superimposed on the “MSY” scenario to better assess the impact of rising temperature on cod and whiting that otherwise showed collapsing biomasses and catches^40,44^ under the “status quo”. Implementing an EAF has been used to investigate fishing policy options providing maximum sustainable yields and to minimise the impact on marine ecosystems^53^. In line with these analyses, our results revealed that sustainable fisheries management can improve the yields of target species in the West Coast of Scotland ecosystem (Fig. 4). However, some MSY estimates, used here as best available proxies, were determined by ICES, over larger areas and multiple stocks (Table 4) and in the case of saithe, large demersals and horse mackerel the sudden changes between F_status quo_ and F_MSY_ caused large changes in predicted catches (Fig. 4). These large variations are unrealistic as annual changes in fishing mortality are moderated to be within ±15%^54,55^. Moreover, we used a constant fishing mortality to 2100, assuming that this level of fishing will be sustainable into the future. However, F_MSY_ values will change as mortality and growth changes in response to temperature-driven changes in community structure. These aspects are obvious limitations of our study. Fishing mortality is revised yearly, based on single species stock assessments related to observed data. Fisheries projections therefore rarely extend beyond 20–30 years, while the impact of rising sea temperature is often assessed on long timescales^56^. The purpose of our study was not to predict what the ecosystem will look like in the future, but to assess the potential impact of rising temperatures for important commercial stocks in an ecosystem harvested at current estimates of F_MSY_. Future work should determine an appropriate target fishing mortality which accounts for the combined effects of fishing temperature by using a more realistic fishing mortality implemented annually through a harvest control rule designed to achieve MSY.

The sensitivity of marine species to temperature changes is defined by thermal tolerance windows that link their habitat temperature within the seasonal variability^36,57^. Within the boreal species investigated here, we identified three groups of stenothermal species. One group, constituted by species with extreme low optimum temperatures (e.g. cod and herring, Fig. 1b) was highly sensitive to relatively small changes in temperature and declines were predicted under all the IPCC scenarios. Only under the “MSY + RCP2.6” scenario, cod biomass increased by 2100 (Fig. 4) associated with the predicted lower temperatures of this climate scenario (Fig. 2) amplified by the AMO “cooling” phase expected by the end of the century (Fig. 6).

The second group consists of boreal species that have low optimum temperatures (e.g. grey seal and haddock, Fig. 1b), showing a steady equilibrium under the best case scenario (RCP2.6), but declining under the three accelerated temperature scenarios (RCP4.0, RCP6.0 and RCP8.5, Fig. 4). A different consideration is required for saithe, which represents the third group: this boreal species had a colder optimum temperature, but a wider thermal tolerance (Fig. 1b). This higher tolerance limit compared to other boreal species allowed saithe to be more resistant to rising temperatures (Fig. 4). A reduction in biomass for this species was predicted only under the warmest climate scenario (RCP8.5) (Fig. 4). The spatial distribution of saithe currently extends south to the English Channel and the Bay of Biscay (http://www.aquamaps.org/)^58^, albeit in smaller abundances. Future applications of this model could be developed to assess a potential deeper spatial distribution for this species.

In contrast to the other boreal gadoids, whiting is a Lusitanian species with southerly geographical distribution from the Iberian Peninsula to as far north as the northern North Sea^59^. Our results showed an increase in whiting biomass under all RCP scenarios, resulting from the effects of higher optimum temperature for this species combined with contemporary declines of its predators such as seals and cod. This result indicated that higher fishing mortalities could be advised for this species if the stock were to recover from the declining historical trends. However, an increase in biomass could lead to decreasing growth and/or recruitment rates due to competition, and a reduction in predation pressure could result in a lower natural mortality, which might produce lower estimates of F_MSY_.

Mackerel, horse mackerel and Norway lobster constituted a group of eurythermal species (Fig. 1b). These species have extensive distributions in temperate latitudes (http://www.aquamaps.org/)^58^, where seasonality in temperature is strong^36,57^. We therefore expect them to be less influenced by the warming climate.

Of the target species in this study, mackerel, horse mackerel and saithe were the dominant species in terms of biomass and catch and were not significantly affected by temperature changes. Under rising temperature scenarios, overall cumulative decreases of biomass and catches of up to 15% and 20% respectively were predicted. However, under the highest scenario, our results suggested that in the long term the large increases predicted for whiting could compensate for the losses predicted in biomass and catches of other species (Fig. 5). Excluding mackerel and horse mackerel, which showed seasonal migration patterns in the study area^45,60^, the observed large increases of saithe and hake in recent years^52^ with an increase of whiting predicted by rising temperature, suggested the possibility of a significant change in the fish community due to both changes in fishing practices and climate change.

The ecosystem model employed here included ecological populations and their interactions within defined spatial distributions. Predicted declines of stocks in this study do not indicate their collapse across the Northeast Atlantic, but rather that they are likely to migrate north or to deeper and cooler waters outside of the modelling study area (WCS, continental shelf of ICES Division VIa). Whilst bathymetric shifts were more difficult to assess^61^, the predicted poleward shift of marine organisms in the European shelf regions have already been observed^62^. The North Sea, an ecosystem comparable to the WCS in terms of latitude and species composition, had an increase in sea surface temperature > 1 °C over the last 40 years, which caused one of the fastest^11^ latitudinal distributional shifts globally for zooplankton^63,64^, fish^65^ and invertebrates^66^, while the combined pressures of a warming climate and fishing produced a strong impact on the distribution and abundance of flatfish^67^ and cod^68^.

Increasing ocean temperatures may put species beyond their tolerance thresholds, causing spatial distribution shifts and producing increases in eurythermal species and the decline of boreal stenothermal species^36,69^. Geographic barriers can also constrain range shifts causing a loss of endemic species^70^. In more extreme cases alien species can colonise new niches adding trophic competition with native species and changing the predator-prey dynamics^69,71,72^. Compositional changes, at the regional scale, could trigger strong potential cascading effects through the tropic web^73^, strengthening and/or weakening trophic relationships to an unprecedented level^61^. Such changes in ecosystem structure will need to be incorporated into the EwE model to address the possible impact that the complete extirpation or introduction of invasive species might have on the ecosystem function and resilience.

The new capability of EwE for using species temperature functions to define species thermal niches^74^ has enabled temperature to be included in the model as an ecosystem driver to assess the impact of warming climate on the food consumption (based on the foraging arena theory^75^). The reliability of these predictions is dependent on the quality of the data used to define the species thermal niches^74^. Nevertheless, it is important to emphasise that this study did not consider potential phenotypic plasticity nor ontogenetic variations of species thermal range, with early stages (e.g., egg and larvae) generally being more sensitive to temperature change^76^. Moreover, shifts of thermal habitat could also lead to species changes of life cycles and new physiological adaptations as already observed for terrestrial ectotherms^77^, aspects which were not taken into consideration in our study. Similarly, the effect that temperature will have on the general metabolism (production and respiration) of species, growth rate, which might increase or decrease size-at-age and have an impact on the total biomass and potential size-structured predator-prey dynamics, were not explicitly included in this model. An increase in temperature might increase the turnover rate (P/B ratio) of some species, and might have an effect on the recruitment of populations, which at present is also not included in this model. These impacts might have unforeseen consequences to the outcome of these simulations and for ecosystem interactions in general, which warrants more study in future. In addition, the use of Monte Carlo simulations after fitting the model, could affect the outcome of the study. If the Monte Carlo simulations were run first and different Ecopath input estimates obtained before the fitting procedure, the estimation of top down and bottom up control of predator-prey dynamics might have changed and this might change the outcome of the future projections. This is also a fruitful area of study for future work.

These caveats notwithstanding, our results showed that the inclusion of temperature tolerances allows for the exploration of different management approaches in a warming ecosystem to identify those strategies that best meet a range of objectives. This is an important step to the implementation of an ecosystem approach to sustainable fisheries management in a warming ecosystem and could improve our short-term management of declining stocks such as cod, whiting and herring. Our results also show that ocean warming could jeopardise the recovery of boreal stenothermal fish species (cod, herring and haddock), causing a reduction of these stocks in the northern temperate ecosystems. While the model presented here cannot, in its present state, be used for tactical short-term decisions due to too many uncertainties relating to the numerous processes it encompasses, it may be useful in EAF to inform managers on likely future long-term trends in biomass and catches under various ‘what if’ scenarios. Similar models could be used to explore various alternative fishing management strategies under a set of climate scenarios to assess the likely outcomes for target species, their prey and predators, but also future ecosystem health, state and structure. The model-driven scenario evaluation approach would (i) inform managers on the best strategy to pursue, depending on the goal, whilst accounting for future climate change and food web effects, (ii) identify potential risks (e.g. prey species depletion) and benefits (commercial species increasing), and (iii) identify and plan for future knock-on effects such as the socio-economic consequences of having to adapt to a changing ecosystem and the resulting change in commercial species composition, and their impacts on the fishing community. Encapsulating food web functioning in the development of integrated models of ecosystems under environmental change will help us to ensure future sustainable exploitation of our marine resources as part of the transition from single-species management to the holistic ecosystem approach to fisheries (EAF).

## Methods

### Ecopath with Ecosim ecosystem modelling

The model was built in Ecopath with Ecosim (EwE) version 6.5 (July 2016). This framework consists of Ecopath, a mass balance model that creates a baseline snapshot of the ecosystem in a given year (1985 in this case), and Ecosim that models the temporal dynamics (1985-2013 in this case). Ecosim models use foraging arena theory^75^ where each predator/prey interaction is defined by vulnerability parameters that affect the predator consumption rate (Equation 1) to describe the top-down and bottom-up controls of the predator/prey interactions. Vulnerability parameters can range between 1 and infinity, with 2 as the default. Vulnerabilities greater than 2 describes top-down control of the predator-prey relationship, where the predator biomass drives the prey mortalities, whilst vulnerabilities between 1-2 define bottom-up control, where the biomass of the predator has little effect on the predation mortality of that prey. For each predator-prey interaction consumption rates, *Q*_*ij*_, are calculated as:

1 $$Qij=\frac{{a}_{ij}\,\ast \,{v}_{ij}\,\ast \,{B}_{i}\,\ast \,{P}_{j}\,\ast \,{T}_{i}\,\ast \,{T}_{j}\,\ast \,{M}_{ij}/{D}_{j}}{{v}_{ij}+{v}_{ij}\,\ast \,{T}_{i}\,\ast \,{M}_{ij}+{a}_{ij}\,\ast \,{M}_{ij}\,\ast \,{P}_{i}\,\ast \,{T}_{j}/{D}_{j}}\,\ast \,f(En{v}_{function},\,t)$$

where *a*_*ij*_ is the effective search rate for predator *j* feeding on a prey *i*, *v*_*ij*_ is vulnerability expressing the rate with which prey *i* move between being vulnerable and not-vulnerable, *B*_*i*_ is prey biomass, *P*_*j*_ is predator biomass (or abundance for split groups), *T*_*i*_ represents prey relative feeding time, *T*_*j*_ is predator relative feeding time, *M*_*ij*_ are the mediation forcing effects, and *D*_*j*_ represents handling time as a limit to consumption rate^75,78^. [*f(Env_function, t)*] is the environmental response function that restricts the size of the foraging arena^75^ to account for external environmental drivers changing over time, such as temperature and salinity.

### Updating and fitting the new West coast of Scotland model

The model boundaries cover the continental shelf of the west coast of Scotland (WCS), an area of approximately 110,000 km^2^ within the International Council for the Exploration of the Sea (ICES) division VIa. The WCS model was updated in both Ecopath and Ecosim (details in Supplementary Methods Tables S1–S6). In this study, we used the new capability of EwE 6.5 (www.ecopath.org) to convert species optimum, minimum and maximum temperatures to species-specific normal probability distributions centred on the species optimum temperature and positive/negative standard deviations defined by the species tolerances (Fig. 1a,b and Supplementary Table S8). The difference between sea temperature and the species thermal optimum distribution was used to scale the consumption rates of each predator: by a factor of 1 at the optimum and declining as the difference from the optimum increases according to Gaussian functions at a rate reflecting the species thermal tolerance range (expressed by the standard deviation of the function) (Fig. 1a).

Fishbase^79^, SeaLifeBase^80^ and AquaMaps^58^ websites provided the species optimum, minimum and maximum temperatures: optimum temperatures were estimated by averaging the 10^th^ and 90^th^ preferable temperature percentiles. For consistency we gathered species optimum temperatures and tolerances data from the same sources except for cod^81^ and kelp^82^ for which local data were used^81^. For functional groups defined by multiple species, the temperature parameters were calculated as geometric means weighted by species biomass or catch composition. Optimum temperatures for juvenile gadoids were raised by 1 °C assuming a shallower distribution in warmer waters to facilitate the growth rates^83^. No local temperature tolerance data were found for phytoplankton, small and large zooplankton; the average of depth-integrated temperature was used for these groups with wide tolerances ranges (Supplementary Table S8).

The model was fitted using an automated stepwise fitting procedure^84^. This procedure searched for different vulnerability parameters and/or numbers of spline points on a primary production anomaly, then calculated the weighted sum of squares (SS) differences between predicted and observed data (Supplementary Table S6) for each iteration. It used the SS and the number of parameters estimated to calculate the Akaike Information Criterion (AIC)^85^, and the corrected AICc^86^. These AIC values were used to assess the model baseline (no ecosystem drivers such as fishing, no primary productivity forcing function and no trophic vulnerabilities applied) and to identify the vulnerability parameters and spline points that produced the lowest AIC. AIC values are also used to calculate Akaike weights which represent the probability that a given model is the best fitted model^87^.

Vulnerability parameters were assumed to be “by predator”, i.e. all iterations assumed the same top-down or bottom-up control of the predator on all of its prey^84^. The PP_anomaly function could represent an environmental driver that can affect primary productivity and is therefore often related to climate variability indices^15,47,88,89^. The fitting procedure was performed with and without temperature and output compared with previous versions of the model fitting^40,84^. The fitting procedure identified the best parameter values that improve the statistical fit of the model. However, as the observed data has its own inherent errors, it is important to analyse a range of model fits with the low AIC values and relevant Akaike weights to identify the best ecologically sensible parameters that best describe the predicted historical trends of the target species^84,90^.

Spearman correlation tests were used to assess collinearity between the predicted primary production anomaly function and environmental temperature (SST) as well as other climate indices such as the smoothed and unsmoothed Atlantic Multi-decadal Oscillation (AMO), and North Atlantic Oscillation Index (NAOI).

### Model uncertainties and validation

Monte-Carlo simulations were performed to investigate the quality of the input data assessing the sensitivity of the best-fitted Ecosim output to uncertainty in the Ecopath basic inputs (B, P/B, Q/B and EE)^78,90^, by assuming a change of 10% in each of these inputs, as well as by using the input pedigree^91^ to describe the uncertainty surrounding the input data for B, Q/B and EE (supplementary Table S4)^90^. In the pedigree-based Monte Carlo simulations the confidence interval of the production/biomass ratio (P/B) was kept at 10% as a cumulative effect of lower confidence intervals for both biomass and P/B determined a large amplification in uncertainty for the Monte Carlo simulations. 200 Monte-Carlo simulation trials for each target functional group in this study were carried out to determine the 5% and 95% confidence interval of the best fitted model (Supplementary Fig. S2).

The model’s capability to predict future observations (model validation) was performed creating three validation datasets (A: 22 years for model fitting, 7-year validation set; B: 24 years for model fitting, 5 year validation set; C: 26 years for model fitting, 3 year validation set). The performances of these 3 models were assessed by comparing the root mean square deviations (RMSD) between predicted and observed values across three validation subsets (Table 3). Over- and underestimation for the target species predictions were visually assessed by plotting predicted vs observed data for both biomasses and catches (Supplementary Fig. S1).

### Historical time series of temperature

Spatial Sea Surface Temperatures (SST) from the Hadley Centre HadISST dataset (http://www.metoffice.gov.uk/hadobs/hadisst/) between 1960–2013 were obtained and annual averages calculated. These results were cross validated using the Millport sea temperature time series previously used to describe the WCS ecosystem^44^. Depth integrated temperature (DIT, black points in Fig. 2) was calculated by scaling the Hadley Centre time series to the difference between surface and near-bottom water obtained from Berx and Hughes^92^ (http://ocean.ices.dk/Project/OCNWES/Default.aspx extracted 15 June 2016). The differences between surface and near-bottom temperatures were homogeneous in space over the UK continental shelf (Supplementary Fig. S3) and therefore an average scaling factor was calculated for the whole continental shelf of ICES VIa (0.61 °C).

### Simulation scenarios

Exploring the effect of future scenarios requires the Ecosim model to reproduce or hindcast the historical observations. Thus, the “status quo” scenario represented the future projections using the model drivers such as fishing mortalities (F_status_quo_) and water temperature set to that of the last year of the historical observed data (2013). The effect of sustainable fishing for the target species was then assessed comparing biomasses and catches of the “status quo” scenario with that of a “maximum sustainable yields” (MSY) scenario which used single-species fishing mortalities at the maximum sustainable yields (F_MSY_) determined by ICES (Table 4). When not available for VIa, F_MSY_ values of neighbouring stocks were taken as best available estimates.

Subsequently we tested the impact of rising temperature under IPCC-RCP scenarios while keeping fishing pressure constant at rates consistent with MSY. Future SST projections were extracted from the Royal Netherlands Meteorological Institute Climate Explorer portal (http://climexp.knmi.nl) within the study area rectangle from the climate changing multi-model global ensemble means for 2.6, 4.5, 6.5 and 8.5 greenhouse gas concentration scenarios (RCP2.6, RCP4.5, RCP6.5 and RCP8.5 scenarios). Thirty-two model outputs, sourced from the Coupled Model Intercomparison Project phase 5 (CMIP5), were extracted for the study area with temperatures fluctuating around the mean by 6–7 °C (Supplementary Fig. S4). The mean projected rates of increase for all of the RCP scenarios (bold colour lines for 2006–2100 in Supplementary Fig. S4) were then applied as anomalies to the observed DIT to predict future temperature. As a calculated mean across thirty-two global model outputs, the SST rates extracted under the RCP scenarios (Supplementary Fig. S4) were de-trended from the impact of climate variability and showed a smaller variability than DIT (black points in Fig. 2). A 3-year moving average of the historical standard deviation (STDEV) was applied to the anomalies to replicate the variability of the historical DIT trend.

The Atlantic Multidecadal Oscillation (AMO) is calculated as an anomaly of the SST and shows a stronger effect in the North Atlantic region^18,20,21,48^. The smoothed/unsmoothed AMO signal (Fig. 6; http://www.esrl.noaa.gov/psd/data/timeseries/AMO/) defined from de-trended patterns of SST variability in the North Atlantic, shows clear positive and negative phases with a frequency of 60–70 years^93^. In 2012 the AMO reached the end of its positive ‘warming’ phase and approached the start of the “cooling phase” (Fig. 6). Thus, to predict the future AMO trend, the smoothed observed pattern was mirrored from 2007 to 2100 to allow the re-occurrences of historical phases at intervals of approximately 60–65 years (between the lowest values of the historical negative phases, Fig. 6). Finally, the AMO projections were added to the temperature projections (DIT + STDEV + AMO) to simulate the effect of this climate index on the future water temperature projections (2014-2100 in Fig. 2).

## Electronic supplementary material

Supplementary Info
