# Supplementary material for: Impact of ocean warming on sustainable fisheries management informs the Ecosystem Approach to Fisheries
Source: Sci Rep. 2017 Oct 18;7:13438. doi: 10.1038/s41598-017-13220-7 (PMC5647405; doi:10.1038/s41598-017-13220-7)
Supplement: Supplementary file 1 — Supplementary Info [file 41598_2017_13220_MOESM1_ESM.pdf]

Impact of ocean warming on sustainable fisheries management informs the Ecosystem Approach to Fisheries

Serpetti, N., Baudron, A.R., Burrows, M.T., Payne, B.L., Helaouët, P., Fernandes, P.G., Heymans, J.J.

Supplementary Methods

The model. The model was built in Ecopath with Ecosim (EwE) version 6.5 (July 2016). Ecopath is a mass balance representing a snapshot of the ecosystem in a given year, 1985 in this case. For each functional group *(i)*, Ecopath requires three of the following four inputs: starting biomasses *(B_i,* t*km^{-2}*)*, production/biomass ratio *(P_i/B_i,* year^{-1}*)*, production/consumption ratio *(Q_i/B_i,* year^{-1}*)*, or ecotrophic efficiency *(EE_i)* which indicates the proportion of mortality explained by the model. In addition, to estimate how the energy is flowing through the ecosystem, trophic relationships within the functional groups (proportion of each prey in the diet) and fishing information (yield= landings + discards, t*km^{-2} year^{-1}) are also required.

Ecosim then uses these inputs as starting information to simulate the dynamics over time of each functional group. In Ecosim a series of differential equations describe changes in biomass for each trophic group (i) over time as:

$$\frac{dB_i}{dt} = \left(\frac{P}{Q}\right)_i \sum_j Q_{ji}\,(t) - \sum_j Q_{ij}\,(t) + I_i - B_i * (M_i + F_i + e_i) \qquad (1)$$

Where $\frac{dB_i}{dt}$ is the biomass growth rate of group *i* in the time dt, $\left(\frac{P}{Q}\right)_i$ is its production/consumption ratio, Q_{ji} is the consumption of group *j* (predator) on prey group(s) *i*, Q_{ij} is the consumption for predation by all predators *j* on group *i* (prey), I_i is the immigration rate, B_i is the starting biomass, M_i and F_i are the natural and fishing mortality rates of group *i*, respectively, and e_i is the density dependent emigration rate[1]. Fishing mortality or fishing effort is used to drive the model and Ecosim predicts biomasses and catches that can then be compared to observed time-series data of biomass and catches using a log-likelihood sum of squares for calibration[2]. For each predator, consumption (Q_{ij}) is calculated based on the "foraging arena" theory[3] which define the degree of "vulnerability (*v*)" of the prey versus its predators due to the assumption that animals can optimise the way they spend their time[4], balancing predation risk with foraging[1].

The study area the EwE model of the West Coast of Scotland (WCS)[5] is defined by ICES VIa division continental shelf (>200 m) covering about 110,000 km^2. The model includes five fleets (demersal trawl, nephrops trawl, other trawl, potting and diving, and pelagic trawl) and a total of 41 functional groups including marine mammals (3), seabirds (1), fish (23, six of which were composed of adult and juvenile stages for cod, haddock and whiting), invertebrates (5), cephalopods (1), zooplankton (2), benthos (3), primary producers (2), and detritus (1) (for details please refer to Alexander et al.[5]. Functional groups were defined by combining species that are similar in predator-prey interactions, ecosystem functioning and habitat preferences[5].

Model updating: Ecopath. The WCS model was updated in both the Ecopath (basic balanced baseline) and Ecosim (temporal dynamics). The hindcasted biomasses and catches for 1985 were updated with the latest data available (stock assessment and ICES DATRAS, http://www.ices.dk/marine-data/data-portals/Pages/DATRAS.aspx): starting biomasses of

mackerel and horse mackerel were divided by 2 to take into account their migratory habits within the ICES Division VIa.
Age-length keys obtained from the ICES DATRAS database were applied to von Bertalanffy growth functions to obtain the basic inputs for the three multi-stanza groups (cod, haddock and whiting). Ecopath biomass basic estimates, landings and discards basic inputs are shown in Supplementary Table S1, S2 and S3 respectively.

Supplementary Table S1. Basic estimates indicating basic inputs (in bold) and estimated values of trophic level, biomass, production/biomass (P/B), consumption/biomass (C/B), ecotrophic efficiency (EE), and production/consumption (P/C).

Group num.	Group name	Trophic level	Biomass (t/km²)	P/B (/year)	C/B (/year)	EE	P/C
1	Grey seals	4.477	0.036	**0.114**	11.388	**0.000**	0.010
2	Harbour seals	4.602	0.011	**0.101**	10.124	**0.000**	0.010
3	Cetaceans	4.237	0.023	0.020	14.000	**0.000**	**0.001**
4	Seabirds	4.145	0.025	0.400	83.051	**0.000**	**0.005**
5	Cod mature	4.019	0.297	1.143	3.500	**0.594**	**0.326**
6	Cod juv.	3.178	0.264	2.207	9.048	**0.845**	**0.244**
7	Haddock mature	3.614	0.563	1.096	4.960	**0.402**	**0.221**
8	Haddock juv.	2.939	0.416	1.743	11.037	**0.889**	**0.158**
9	Whiting mature	4.157	0.390	1.131	4.500	**0.581**	**0.251**
10	Whiting juv.	3.045	0.396	1.710	9.016	**0.916**	**0.190**
11	Saithe	3.971	0.505	**0.937**	4.686	**0.612**	0.200
12	Gurnards	3.604	**0.123**	**0.824**	4.122	0.950	0.200
13	Monkfish	4.323	**0.094**	**0.480**	1.714	0.950	0.280
14	Flatfish	3.413	**0.740**	**1.130**	3.768	0.950	0.300
15	Rays	3.858	**0.119**	**0.449**	2.243	0.950	0.200
16	Sharks	4.066	**0.242**	**0.682**	3.410	0.950	0.200
17	Large demersals	4.274	**1.034**	**0.488**	2.442	0.950	0.200
18	Benthopelagic fish	3.245	**0.601**	**1.581**	5.270	0.950	0.300
19	Mackerel	3.340	4.319	0.626	4.400	**0.649**	**0.142**
20	Horse Mackerel	3.187	3.310	**0.740**	3.700	**0.718**	0.200
21	Blue Whiting	3.635	1.978	1.500	**6.000**	**0.603**	0.250
22	Other pelagics	3.516	**2.123**	**1.800**	6.000	0.950	0.300
23	Herring	3.156	5.943	1.500	10.100	**0.694**	**0.149**
24	Norway pout	3.276	**1.292**	**1.680**	5.600	0.950	0.300
25	Poor cod	3.530	**0.072**	**1.170**	3.900	0.950	0.300
26	Sandeel	3.184	**1.228**	**1.826**	6.085	0.950	0.300
27	Sprat	3.159	**1.758**	**1.584**	5.280	0.950	0.300
28	Norway lobster	3.415	1.000	0.730	4.876	**0.482**	**0.150**
29	Lobster	3.395	**0.029**	0.338	3.650	0.950	**0.093**
30	Edible crab	3.324	**2.497**	0.354	**2.360**	0.950	0.150
31	Velvet crab	2.622	**0.808**	0.646	12.775	0.950	**0.051**
32	Crustaceans	2.691	**14.012**	0.871	5.807	0.950	**0.150**
33	Cephalopod	3.241	**0.982**	1.981	15.000	0.950	**0.132**
34	Large zooplankton	2.158	**14.775**	10.000	35.000	0.950	**0.286**
35	Small zooplankton	2.031	**7.821**	18.000	72.000	0.950	**0.250**
36	Infauna	2.037	**3.048**	20.000	80.000	0.950	**0.250**
37	Scallops	2.000	**9.375**	0.445	14.334	0.950	**0.031**
38	Epifauna	2.391	**2.813**	20.000	80.000	0.950	**0.250**
39	Algae	1.000	**5.743**	5.000	0.000	0.950	
40	Phytoplankton	1.000	**16.569**	70.000	0.000	0.950	
41	Detritus	1.000	100.000			**0.861**	

Supplementary Table S2. Basic inputs of landings data for the functional groups for each fleet[5]: DTR (demersal trawling), NTR (nephrops trawling), OTR (other trawling), POT (potting and diving), PTR (pelagic trawling).

Group	Group	DTR	NTR	OTR	POT	PTR
1	Grey seals	0	0	0	0	0
2	Harbour seals	0	0	0	0	0
3	Cetaceans	0	0	0	0	0
4	Seabirds	0	0	0	0	0
5	Cod mature	0.146	0.015	$8.72*10^{-5}$	0	0
6	Cod juv.	0.006	0.001	$5.84*10^{-6}$	0	0
7	Haddock mature	0.145	0.004	$1.05*10^{-5}$	0	0
8	Haddock juv.	0.0007	$9.41*10^{-6}$	$3.54*10^{-7}$	0	0
9	Whiting mature	0.098	0.012	$2.58*10^{-7}$	0	0
10	Whiting juv.	0.005	0.002	$2.61*10^{-8}$	0	0
11	Saithe	0.204	0.024	0.0002	0	0
12	Gurnards	0.002	0.0001	0	0	0
13	Monkfish	0.029	0.008	$1.59*10^{-5}$	0	0
14	Flatfish	0.046	0.013	$9.42*10^{-5}$	0	0
15	Rays	0.028	0.005	0.0002	0	0
16	Sharks	0.096	0.022	0.0008	0	0.003
17	Large demersals	0.173	0.042	0.0002	0	0
18	Benthopelagic fish	0.002	0.0002	$2.48*10^{-5}$	$8.64*10^{-5}$	0.0016
19	Mackerel	0.0002	0.007	0	0	1.011
20	Horse Mackerel	0.0003	0.0003	0.173	0	0.127
21	Blue Whiting	0.002	0.011	0.0015	0	0.861
22	Other pelagics	0	0	0	0	0
23	Herring	0.0002	0.0007	$6.01*10^{-5}$	0	0.357
24	Norway pout	0	0	0.0105	0	0
25	Poor cod	0	0	0	0	0
26	Sandeel	0	1.04E-06	0.169	0	0
27	Sprat	0	0	0	0	0.032
28	Norway lobster	0.007	0.0939	$2.53*10^{-5}$	0.012	0
29	Lobster	$6.1*10^{-6}$	0	$4.16*10^{-5}$	0.0056	0
30	Edible crab	$4.77*10^{-5}$	0	0.0003	0.044	0
31	Velvet crab	$1.94*10^{-5}$	0	0.0019	0.002	0
32	Crustaceans	$4.26*10^{-6}$	0	$2.9*10^{-5}$	0.004	0
33	Cephalopod	0.0003	$3.00*10^{-5}$	$2.12*10^{-7}$	0	0
34	Large zooplankton	0	0	0	0	0
35	Small zooplankton	0	0	0	0	0
36	Infauna	0	0	0	0	0
37	Scallops	$2.01*10^{-5}$	$2.13*10^{-5}$	0.038	0.022	0
38	Epifauna	$5.48*10^{-6}$	$5.80*10^{-6}$	0.0103	0.006	0
39	Algae	0	0	0	0	0
40	Phytoplankton	0	0	0	0	0
41	Detritus	0	0	0	0	0

Supplementary Table S3. Basic inputs of discard data for the functional groups for each fleet[5]: DTR (demersal trawling), NTR (nephrops trawling), OTR (other trawling), POT (potting and diving), PTR (pelagic trawling).

Group num.	Group name	DTR (t/km²/year)	NTR (t/km²/year)	OTR (t/km²/year)	POT (t/km²/year)	PTR (t/km²/year)
5	Cod mature	0.0003	$3.40*10^{-5}$	$1.94*10^{-7}$	0	0
6	Cod juv.	0.007	0.0015	$6.35*10^{-6}$	0	0
7	Haddock mature	0.032	0.0009	$2.31*10^{-6}$	0	0
8	Haddock juv.	0.011	0.0092	$5.71*10^{-7}$	0	0
9	Whiting mature	0.025	0.0028	$5.99*10^{-8}$	0	0
10	Whiting juv.	0.0112	0.005	$6.67*10^{-8}$	0	0
12	Gurnards	0	0	0	0	0
13	Monkfish	0.0018	0.0005	$9.80*10^{-7}$	0	0
14	Flatfish	0.013	0.0036	$2.66*10^{-5}$	0	0
15	Rays	0.006	0.0012	$4.85*10^{-5}$	0	0
16	Sharks	0	0	0	0	0
17	Large demersals	0.0455	0.0111	$4.71*10^{-5}$	0	0
18	Benthopelagic fish	0.0003	$3.43*10^{-5}$	$4.00*10^{-6}$	$1.40*10^{-5}$	0.00026

Adjusting the diet matrix is a powerful and often underused way of improving EwE models[6]. Thus, the original diet matrix[7] was revisited based on updated knowledge of stomach contents from DAPSTOM database[8] and from two closely related ecosystems, the North Sea[9] and Irish Sea[10], assuming that species on the WCS consume similar prey as in those adjacent ecosystems. Please for Ecopath diet matrix details refer to Supplementary Table S5. To ecologically test the model mass-balance of the updated Ecopath model, a pre-balance (PREBAL) analysis[11] was applied and compared with the previous WCS model[5,12]: no substantial differences were found (data not shown). Confidence interval of the data inputs (pedigree) was also assessed (Supplementary Table S4) for subsequent analysis of the prediction uncertainties.

Supplementary Table S4. Input data pedigree indicating confidence interval (%) of the data input. The highest confidence interval (80%) was allocated when the data are estimated by Ecopath, sourced from other model or guess estimation, 50% where input data were calculated by indirect methods, 30% when input data were sampled in the study area with a low precision and 10% with high precision.

Group	Group name	Biomass	Production /	Consumption /	Diet	Catch
1	Grey seals	50	80	50	10	NA
2	Harbour seals	50	80	50	10	NA
3	Cetaceans	50	80	80	80	NA
4	Seabirds	50	80	80	80	NA
5	Cod mature	10	50	50	30	50
6	Cod immature	10	80	80	30	50
7	Haddock mature	50	50	50	30	50
8	Haddock immature	50	80	80	30	50
9	Whiting mature	10	50	50	30	50
10	Whiting immature	10	80	80	30	50
11	Saithe	50	80	50	30	50
12	Gurnards	80	80	50	80	50
13	Monkfish	80	80	50	30	50
14	Flatfish	80	80	50	30	50
15	Rays	80	80	50	80	50
16	Sharks	80	80	50	80	50
17	Large demersals	80	80	50	30	50
18	Benthopelagic fish	80	80	50	80	50
19	Mackerel	50	50	50	30	50
20	Horse Mackerel	50	80	50	30	50
21	Blue Whiting	50	50	80	30	50
22	Other pelagics	80	80	50	80	50
23	Herring	10	50	50	30	50
24	Norway pout	80	80	50	30	50
25	Poor cod	80	80	50	30	NA
26	Sandeel	80	80	50	30	50
27	Sprat	80	80	50	30	50
28	Nephrops	50	80	80	80	50
29	Lobster	80	50	50	80	50
30	Edible crab	80	50	80	80	50
31	Velvet crab	80	50	50	80	50
32	Crustaceans	80	80	80	80	50
33	Cephalopod	80	80	80	80	50
34	Large zooplankton	80	80	80	80	NA
35	Small zooplankton	80	80	80	80	NA
36	Infauna	80	80	80	80	NA
37	Scallops	80	50	50	80	50
38	Epifauna	80	80	80	80	50
39	Algae	80	NA	NA	NA	NA
40	Phytoplankton	80	NA	NA	NA	NA
41	Detritus	NA	NA	NA	NA	NA

Model updating: Ecosim. Observed biomass time-series data were obtained from ICES DATRAS following the method from Baudron and Fernandes [13] for demersal and benthic groups whilst assessment data were used for gadoid multi-stanza groups[14] and underwater TV surveys[14] for Norway lobster species. Pelagic species are not effectively captured by bottom

trawl surveys. Therefore, when available, other biomass data sources such as acoustic surveys for the subarea VIa north for herring[15] and total stock biomass estimates for the western shelf (scaled down to VIa using the average proportion of landings) for mackerel and horse mackerel[16] were used. Biomass estimates of grey and harbour seal pup production from the Inner and Outer Hebrides[17] were used as biomass trends for these species.

Abundances values of small (< 2 mm) and large (> 2 mm) zooplankton, and phytoplankton Colour Index (PCI) were obtained from the Sir Alister Hardy Foundation for Ocean Science (SAHFOS). The PCI constitutes a semi-quantitative representation of the total phytoplankton biomass. PCI is a rough assessment of the greenness of the Continuous Plankton Recorder (CPR) silk into 5 categories that consistently reflects not only changes in the abundance of phytoplankton, but also its composition[18]. Multi-scale temporal eigenfunction analysis, namely distance-based Moran's eigenvector maps (dbMEM[19], was applied on planktonic data. The decomposition of the temporal relationships among plankton into all possible scales of variation along the time series allowed the calculation of a monthly time series broad-scale submodel.

Catch time-series for gadoid multi-stanza groups were obtained from stock assessment reports which accounted for discards. Cod and whiting are assessed in ICES Division VIa, however haddock is assessed for both IV and VIa areas combined[20], hence, ICES VIa was assumed to contribute 9.5% of haddock catches as this is the proportion of haddock TAC allocated to VIa[21]. For all other groups, 1985-2013 landings were obtained from ICES standardised surveys (STATLANT, http://ices.dk/marine-data/dataset-collections/Pages/Fish-catch-and-stock-assessment.aspx). Discard rates have only been recorded since 2003[22], thus 1985-2002 discard rates were estimated by inversely applying 2003-2013 average discard rates to 1985-2002 landings time-series. Standardised surveys well represented temporal trends, but biomass values are underestimated compare to stock assessments estimates. Biomass time-series obtained from these datasets were therefore raised by the 1985 starting biomass values before calculating the exploitation rate or fishing mortality (F) as catch to biomass ratio (C/B). Lastly, the "feeding time adjustment rate" (Dj in equation 4) was set to 0.5 for mammals as suggested by Christensen, et al. [1] and to 0.2 for immature stanzas which still feed on egg content in early life stages, while it was set to 0 for all other groups. Ecosim time-series are shown in Supplementary Table S6.

Time-series of depth integrated temperature (DIT) (Fig. 2 and Fig. 3c) were added to the model as an absolute forcing function and functional groups optimum temperatures and tolerances (defined by left/right standard deviations of the response function) were used to calculate the group functional responses to temperature (Supplementary Table S8).

Model updating: fitting. The model fitting was performed using an automated stepwise fitting procedure[23] was used to define the ecosystem driving forces (e.g. fishing), the vulnerability values of the trophic interactions as well as a primary productivity anomaly function that improve the statistical fit of predicted/observed data using the weighted sum of squared differences (SS) and the Akaike Information Criterion (AIC). The stepwise fitting procedure tested 1,990 model interactions based on 28 time-series of relative biomasses, 22 time-series of catches and 22 of fishing mortality and 9 time-series of forced catches with a total of 1,356 observations (observed data points) estimating a maximum number of 49 parameters (based only on independent time-series). The fitting procedure searched for vulnerability parameters "by predator" for all iterations assuming the same top-down or bottom up control of the predator on all its prey[23]. Fitted vulnerabilities values are shown in Supplementary Table S7.

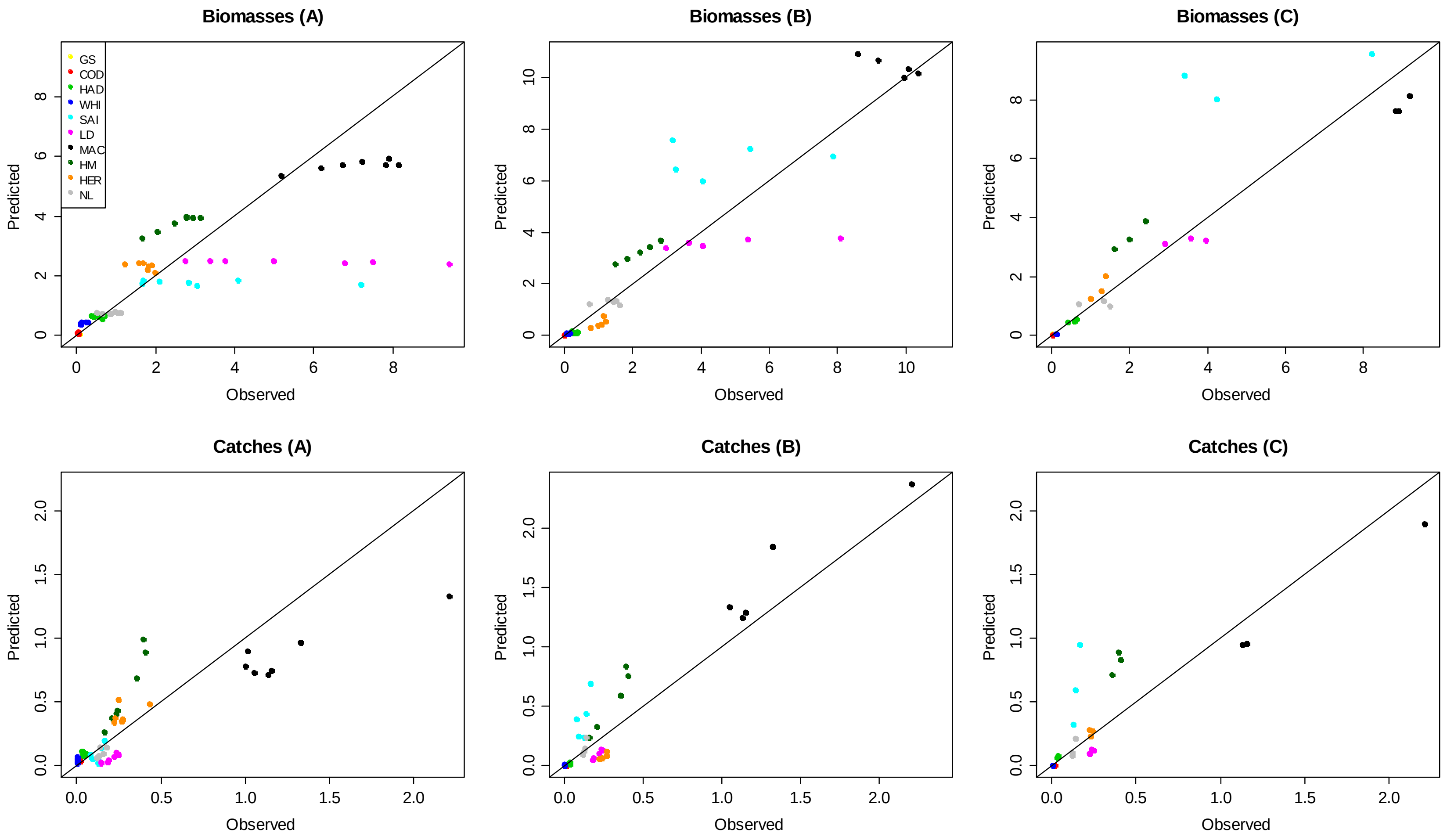

Supplementary Fig. S1. Predicted vs observed data for both biomasses and catches across three validation datasets (A: 22 years for model fitting, 7 years validation set; B: 24 years for model fitting, 5 years validation set; C: 26 years for model fitting, 3 years validation set) illustrating over- and underestimation for the target species predictions (GS (yellow)= grey seals; COD (red)= cod; HAD (light green)= haddock; WHI (blue)= whiting; SAI (light blue)= saithe; LD (magenta)= large demersal; MAC (black)= mackerel; HM (dark green)= horse mackerel; HER (orange)= herring; NL (grey)= Norway lobster).

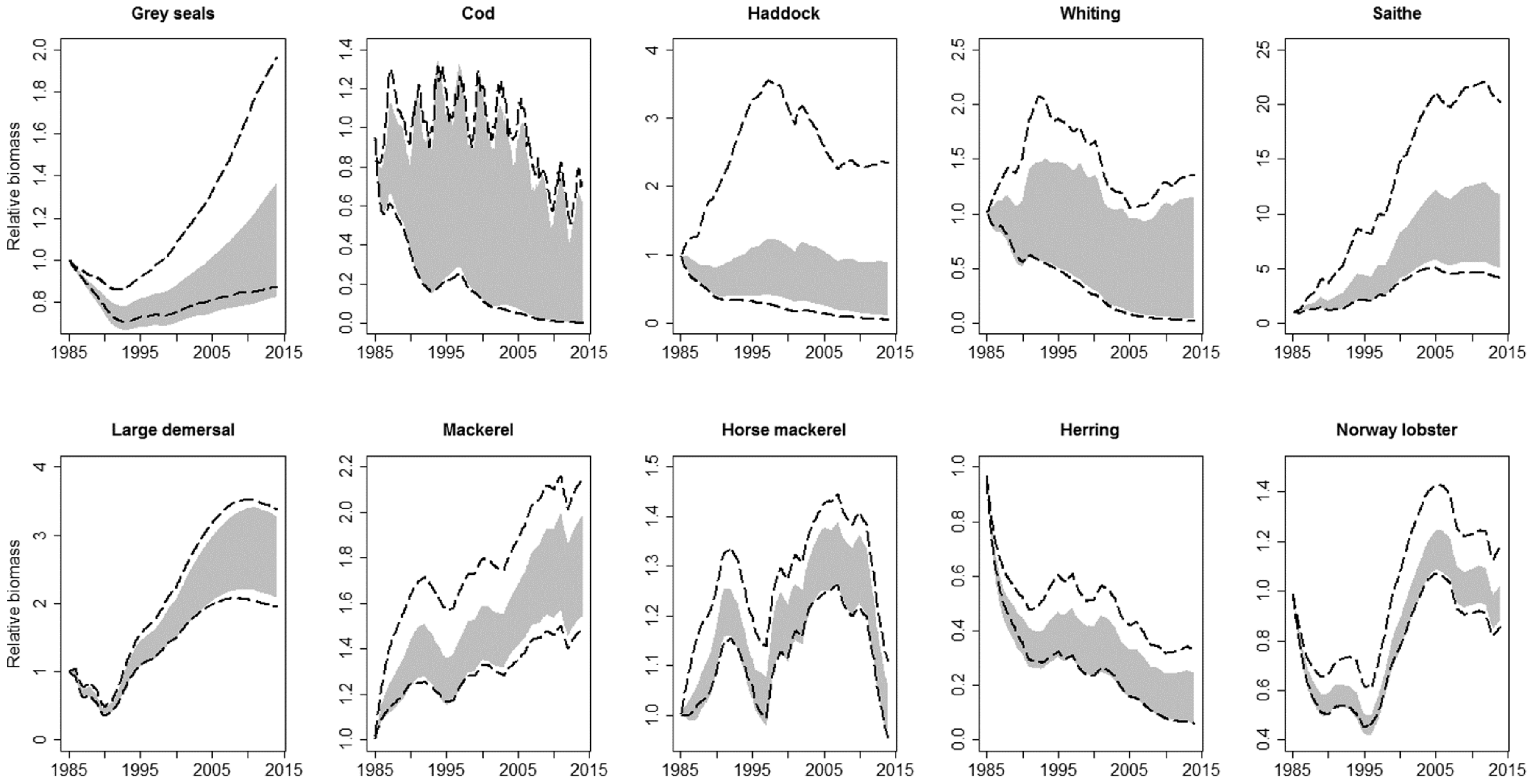

Supplementary Fig. S2. 95% and 5% percentiles of the Monte Carlo simulations using 10% fixed variability of the model inputs (grey area) and the input pedigree (Supplementary Table S4) (dashed lines).

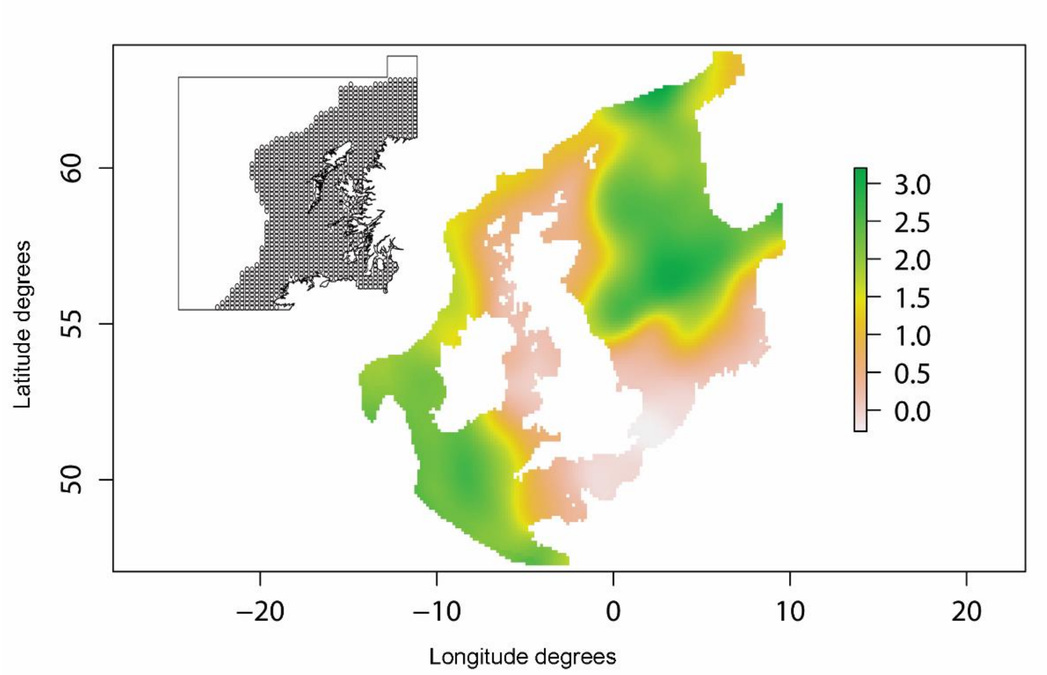

Supplementary Fig. S3. High resolution spatial difference between surface/near-bottom water temperatures in UK waters integrated over 1971-2000. The scaling factor (0.61 °C) was calculated as an average within the continental shelf of the ICES VIa rectangle. Monthly mean data for surface and near-bottom temperatures at ~10km resolution for UK waters can be requested from the authors[24] or be found online at the ICES Ocean climatology of the North-West European shelf, ICES, Copenhagen. (http://ocean.ices.dk/Project/OCNWES/Default.aspx - extraction 15 June 2016). The figure was developed using R software[25] version 3.2.0.

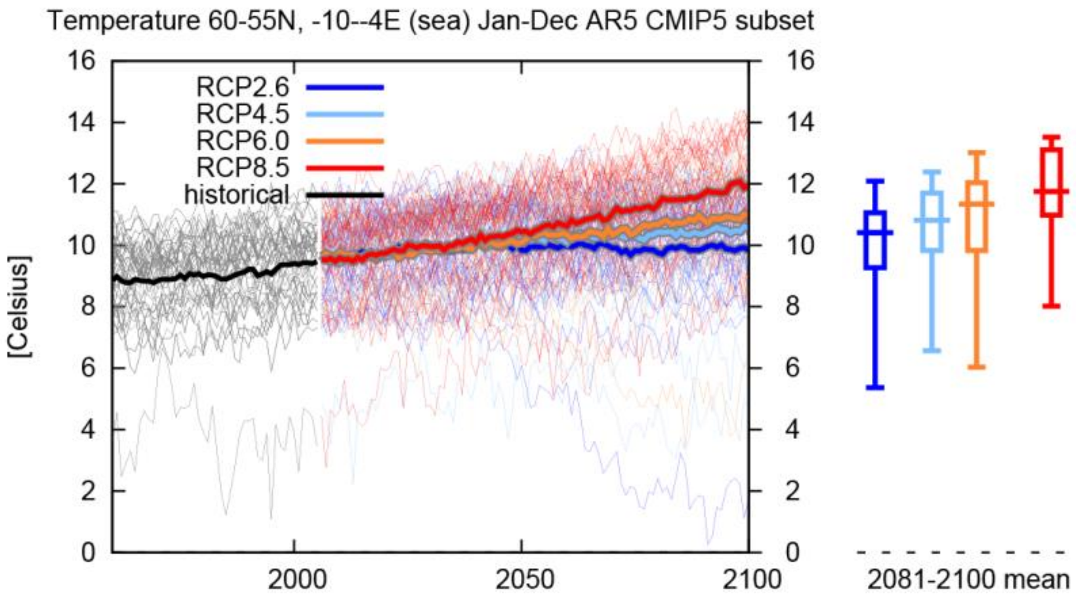

Supplementary Fig. S4. Future SST projections were extracted from the Royal Netherlands Meteorological Institute Climate Explorer portal (http://climexp.knmi.nl) within the study area rectangle from the climate changing multi-model ensemble means (RCP2.6, RCP4.5, RCP6.5 and RCP8.5 scenarios). The RCP scenarios are based on different rates of greenhouse gas emissions by 2100 and consequently rising sea surface temperature (SST) between 2014-2100 in the WCS ecosystem. The RCP2.6 represents a scenario where total radiative forcing is reduced from values of ~3.1 Wm^{-2} in the mid-century to a stabilised value of ~2.6Wm^{-2} by 2100. Even in this scenario, SST is predicted to increase of ~0.5°C by 2100. The RCP4.5 represents a scenario where total radiative forcing is stabilized at~4.5Wm^{-2} by 2100 and in which SST rising of ~1°C by 2100. The RCP6.0 represents a scenario where total radiative forcing is stabilized at~6.0Wm^{-2} with predicted oglobal ocean temperature rising of 1.5°C by 2100. RCP8.5 represents a rising pathway scenario characterized by a continuous increasing of greenhouse gas emission with rates greater than >8.5Wm^{-2} by 2100. RCP8.5 yields the highest rates of warming with global mean sea surface temperature in 2100 increasing by ~2.5°C. Thirty-two model outputs, sourced from the Coupled Model Intercomparison Project phase 5 (CMIP5), were extracted for the study area with temperatures fluctuating around their mean by 6-7 °C.

References

1 Christensen, V., Walters, C. J., Pauly, D. & Forrest, R. Ecopath with Ecosim, version 6. User Guide. 235 pp. (University of British Columbia, Vancouver, B.C., Canada, 2008).

2 Christensen, V. & Walters, C. J. Ecopath with Ecosim: methods, capabilities and limitations. *Ecol. Model.* **172**, 109-139 (2004).

3 Ahrens, R. N. M., Walters, C. J. & Christensen, V. Foraging arena theory. *Fish Fish.* **13**, 41-59 (2012).

4 Walters, C., Christensen, V. & Pauly, D. Structuring dynamic models of exploited ecosystems from trophic mass-balance assessments. *Rev. Fish Biol. Fish.* **7**, 139-172 (1997).

5 Alexander, K. A. *et al.* Investigating the recent decline in gadoid stocks in the west of Scotland shelf ecosystem using a foodweb model. *ICES J. Mar. Sci.* **72**, 436-449 (2015).

6 Ainsworth, C. H. & Walters, C. J. Ten common mistakes made in Ecopath with Ecosim modelling. *Ecol. Model.* **308**, 14-17 (2015).

7 Bailey, N. *et al.* The West of Scotland Marine Ecosystem: A Review of Scientific Knowledge. *Marine Scotland Science Report* **0911** (2011).

8 Pinnegar, J. K., Tomczak, M. T. & Link, J. S. How to determine the likely indirect food-web consequences of a newly introduced non-native species: A worked example. *Ecol. Model.* **272**, 379-387 (2014).

9 Mackinson, S. & Daskalov, G. An ecosystem model of the North Sea to support an ecosystem approach to fisheries management: description and parameterisation. *Cefas Science Series Technical Report* **142** (2007).

10 Lees, K. & Mackinson, S. An Ecopath model of the Irish Sea: ecosystems properties and sensitivity analysis. *Cefas Science Series Technical Report* **138** (2007).

11 Link, J. S. Adding rigor to ecological network models by evaluating a set of pre-balance diagnostics: A plea for PREBAL. *Ecol. Model.* **221**, 1580-1591 (2010).

12 Heymans, J. J. *et al.* Best practice in Ecopath with Ecosim food-web models for ecosystem-based management. *Ecol. Model.* **331**, 173-184 (2016).

13 Baudron, A. R. & Fernandes, P. G. Adverse consequences of stock recovery: European hake, a new "choke" species under a discard ban? *Fish Fish.* **16**, 563-575 (2015).

14 ICES. Report of the Working Group on Celtic Seas Ecoregion (WGCSE) - ICES CM 2014/ACOM:12. 2032 pp. (Copenhagen, Denmark, 2015a).

15 ICES. Report of the Herring Assessment Working Group for the Area South of 62ºN (HAWG)-ICES CM 2014/ACOM:06. 1257 pp. (ICES HQ, Copenhagen, Denmark, 2014b).

16 ICES. Report of the Report of the Working Group on Widely Distributed Stocks (WGWIDE) - ICES CM 2014/ACOM:15. 938 pp. (ICES Headquarters, Copenhagen, Denmark, 2014a).

17 SCOS. Advice on matters related to the management of seal populations. 161pp. (2014).

18 Batten, S. D. *et al.* CPR sampling: the technical background, materials and methods, consistency and comparability. *Prog. Oceanogr.* **58**, 193-215 (2003).

19 Legendre, P. & Legendre, L. in *Numerical ecology* Vol. Chapter 14 (Elsevier Science BV, 2012).

20 ICES. Report of the Working Group for the Assessment of Demersal Stocks in the North Sea and Skagerrak (WGNSSK) - ICES CM 2014/ACOM:13. 1493 pp. (ICES HQ, Copenhagen, Denmark, 2015b).

21 European-Union. *Agreed record of fisheries consultations between the European Union and Norway for 2015*, <https://ec.europa.eu/fisheries/sites/fisheries/files/docs/body/2015-agreed-record-eu-norway-skagerrak_en.pdf> (2014).

22 STECF. Evaluation of Fishing Effort Regimes in European Waters - Part 1 (STECF-14-12). 480 pp. (Publications Office of the European Union, Luxembourg, 2014).

23 Scott, E., Serpetti, N., Steenbeek, J. & Heymans, J. J. A Stepwise Fitting Procedure for automated fitting of Ecopath with Ecosim models. *SoftwareX*, doi:http://dx.doi.org/10.1016/j.softx.2016.02.002 (2016).

24 Berx, B. & Hughes, S. L. Climatology of surface and near-bed temperature and salinity on the north-west European continental shelf for 1971-2000. *Cont. Shelf Res.* **29**, 2286-2292 (2009).

25 R: A language and environment for statistical computing. R Foundation for Statistical Computing (URL http://www.R-project.org/, Vienna, Austria, 2015).

26 Kaschner, K. *et al.* in *AquaMaps: Predicted range maps for aquatic species* Vol. Version 08/2016 (World wide web electronic publication, www.aquamaps.org, 2016).

27 Palomares, M. L. D. & Pauly, D. in *SeaLifeBase* (World Wide Web electronic publication (02/2017), 2017).

28 Froese, R. & Pauly, D. in *Fishbase* (World Wide Web electronic publication (02/2017), 2017).

29 ICES. Spawning and life history information for North Atlantic cod stocks. Report No. 274, 152 pp. (2005).

30 Björnsson, B., Steinarsson, A. & Oddgeirsson, M. Optimal temperature for growth and feed conversion of immature cod (Gadus morhua L.). *ICES J. Mar. Sci.* **58**, 29-38 (2001).

31 Burrows, M. T., Twigg, G., Mieszkowska, N. & Harvey, R. Marine Biodiversity and Climate Change (MarClim) Scotland 2014/15. (Scottish Natural Heritage Commissioned Report No., in press., 2016).

Supplementary Table S5. Revised diet matrix

Group num.	1	2	3	4	5	6	7	8	9	10	11	12	13	14	15	16	17	18	19
1-Grey seals																			
2-Harbour seals																			
3-Cetaceans																			
4 Seabirds																			
5 Cod mature	0.055	0.038	0.002		0.010								0.009						
6-Cod juv.	0.045			0.027	0.077	0.009			0.008	0.005	0.052	0.012	0.156	0.002		0.021			0.005
7-Haddock mature	0.030	0.115	0.000		0.021						0.002		0.059			0.006			
8-Haddock juv				0.006	0.015	0.024	0.000	0.004	0.006	0.002	0.037	0.002	0.059	0.001	0.009	0.001	0.001		0.007
9-Whiting mature	0.027	0.229	0.001		0.045				0.002		0.006		0.052			0.010			
10-Whiting juv.				0.003	0.047	0.023			0.034	0.007	0.030	0.002	0.052	0.001	0.033	0.020	0.030		0.007
11-Saithe	0.060		0.001	0.007					0.007				0.033	0.001		0.001	0.001		
12-Gurnards					0.002		0.003						0.002	0.004	0.005	0.020			
13- Monkfish					0.001								0.010		0.001	0.001			
14-Flatfish	0.096	0.005			0.039				0.001		0.029	0.001	0.063	0.004	0.041	0.010	0.037		
15- Rays					0.001								0.001		0.001	0.010			
16-Sharks			0.000													0.042			
17-Large demersals	0.118	0.097	0.022														0.056		
18- Benthopelagic fish	0.010		0.007	0.030	0.000		0.039					0.030	0.098	0.010		0.024	0.070	0.050	0.006
19-Mackerel		0.018	0.001	0.030	0.006				0.126				0.010	0.005		0.017	0.164		
20-Horse Mackerel		0.346		0.013					0.014		0.026					0.010	0.100		0.020
21-Blue Whiting	0.010			0.032	0.019						0.034						0.171		
22-Other pelagics			0.638	0.125	0.107		0.018		0.123		0.012	0.015	0.023	0.038	0.010		0.081		0.010
23-Herring	0.054	0.077	0.001	0.356	0.092		0.001		0.346		0.147	0.031	0.108		0.130	0.301	0.080		0.100
24-Norway pout	0.038	0.054		0.020	0.015		0.009		0.020		0.201	0.004	0.113	0.015	0.010	0.002	0.010		0.010
25-Poor cod	0.010		0.050	0.001	0.000		0.001		0.001		0.000	0.001	0.001		0.000	0.001	0.001		0.001
26-Sandeel	0.438	0.012	0.077	0.086	0.044	0.040	0.030		0.051	0.020	0.030	0.084	0.104	0.020	0.050	0.021	0.001		0.010
27-Sprat	0.010		0.005	0.135	0.001		0.080		0.133		0.090	0.006			0.040	0.098	0.010		0.010
28-Norway lobster					0.014									0.021	0.130				

29-Lobster					0.001				0.001						0.001				
30-Edible crab					0.006				0.001			0.001		0.001	0.005	0.001			
31-Velvet crab					0.001				0.001						0.001	0.001			
32-Crustaceans			0.002	0.035	0.276	0.050	0.370	0.050	0.003	0.050	0.115	0.431	0.001	0.106	0.249	0.110	0.038	0.004	0.010
33 Cephalopod		0.010	0.004		0.018				0.030		0.005		0.045	0.007		0.120	0.033		
34-Large zooplankton			0.119	0.020		0.353		0.503	0.020	0.445	0.183	0.223		0.100		0.041	0.004	0.648	0.698
35 Small zooplankton			0.070			0.250		0.151		0.201				0.102				0.000	0.100
36-Infauna					0.074	0.150	0.116	0.050		0.100		0.056		0.214	0.040	0.000	0.000	0.110	0.005
37-Scallops					0.001		0.001		0.001			0.002		0.001	0.001				
38-Epifauna			0.000	0.071	0.066	0.050	0.331	0.040	0.071	0.045		0.099		0.346	0.243	0.110	0.112	0.188	
39-Algae																			
40-Phytoplankton						0.050		0.201		0.125									
41-Detritus																			

Continue…

Group num.	20	21	22	23	24	25	26	27	28	29	30	31	32	33	34	35	36	37	38
1-Grey seals																			
2-Harbour seals																			
3-Cetaceans																			
4 Seabirds																			
5 Cod mature																			
6-Cod juv.																			
7-Haddock mature																			
8-Haddock juv	0.014		0.003											0.004					
9-Whiting mature																			
10-Whiting juv.	0.007																		
11-Saithe																			
12-Gurnards			0.002											0.002					
13- Monkfish																			

14-Flatfish			0.002			0.001					0.031			0.015					
15- Rays																			
16-Sharks																			
17-Large demersals																			
18- Benthopelagic fish					0.002	0.111							0.001	0.005					
19-Mackerel																			
20-Horse Mackerel			0.030											0.019					
21-Blue Whiting		0.020												0.005					
22-Other pelagics		0.150	0.034											0.002					
23-Herring	0.016	0.050	0.030											0.027					
24-Norway pout	0.016	0.070	0.005											0.004					
25-Poor cod					0.001	0.001							0.000	0.001					
26-Sandeel		0.010	0.027	0.000		0.002								0.019					
27-Sprat		0.100	0.007	0.000		0.001								0.004					
28-Norway lobster			0.001			0.031							0.001	0.002					
29-Lobster										0.008									
30-Edible crab									0.100	0.001	0.050								
31-Velvet crab									0.100	0.005									
32-Crustaceans	0.010	0.010	0.010	0.010	0.193	0.300	0.050	0.050	0.100	0.320	0.196	0.253	0.010	0.002					
33 Cephalopod			0.122			0.001													
34-Large zooplankton	0.712	0.590	0.189	0.983	0.752	0.097	0.948	0.750	0.199				0.084	0.592	0.012				
35 Small zooplankton	0.215		0.034			0.004	0.001	0.200	0.161	0.003			0.105	0.273	0.140	0.030			0.098
36-Infauna	0.009		0.136			0.161	0.001		0.051	0.059			0.133	0.020			0.036		0.149
37-Scallops						0.001					0.001	0.001	0.020						0.010
38-Epifauna			0.368		0.052	0.290			0.289	0.547	0.576	0.139	0.220	0.004					0.090
39-Algae										0.057	0.010	0.456							0.100
40-Phytoplankton				0.007									0.100		0.710	0.800	0.499	0.500	0.378
41-Detritus											0.136	0.151	0.326		0.138	0.170	0.465	0.500	0.175

Supplementary Table S6. Time series: including 28 time-series of relative biomasses (type= 0), 22 time-series of catches (type= 6), 9 time-series of forced catches (type= -6) and 22 of fishing mortality (type= 4) with a total of 1,356 observations

Group num.	Grey seals	Harbour seals	Cod mature	Cod mature	Cod mature	Cod juv.	Cod juv.	Cod juv.	Haddock mature	Haddock mature	Haddock mature	Haddock juv.	Haddock juv.	Haddock juv.	Whiting mature	Whiting mature	Whiting mature	Whiting juv.	Whiting juv.	Whiting juv.
Type	0	0	6	4	0	6	4	0	6	4	0	6	4	0	6	4	0	6	4	0
1985	0.014	0.011	0.162	0.545	0.297	0.016	0.061	0.264	0.182	0.323	0.563	0.021	0.050	0.416	0.138	0.354	0.390	0.023	0.059	0.396
1986	0.015		0.102	0.468	0.219	0.007	0.010	0.738	0.168	0.493	0.341	0.015	0.015	0.961	0.092	0.265	0.347	0.009	0.026	0.367
1987	0.016		0.134	0.527	0.255	0.060	0.054	1.104	0.134	0.466	0.287	0.026	0.042	0.621	0.127	0.335	0.379	0.086	0.194	0.445
1988	0.015		0.181	0.528	0.343	0.008	0.023	0.343	0.142	0.402	0.354	0.005	0.026	0.193	0.155	0.423	0.365	0.022	0.208	0.107
1989	0.016		0.143	0.589	0.243	0.032	0.045	0.706	0.095	0.485	0.197	0.009	0.032	0.282	0.090	0.406	0.222	0.032	0.080	0.396
1990	0.017		0.108	0.465	0.232	0.008	0.078	0.100	0.058	0.488	0.118	0.013	0.018	0.758	0.062	0.202	0.308	0.030	0.100	0.302
1991	0.019		0.091	0.531	0.171	0.014	0.042	0.348	0.049	0.476	0.104	0.030	0.031	0.974	0.081	0.312	0.260	0.024	0.086	0.278
1992	0.022		0.075	0.494	0.152	0.024	0.049	0.484	0.092	0.405	0.228	0.018	0.023	0.796	0.084	0.307	0.273	0.055	0.129	0.426
1993	0.022		0.092	0.435	0.212	0.003	0.009	0.338	0.119	0.389	0.306	0.028	0.047	0.602	0.089	0.247	0.359	0.017	0.046	0.365
1994	0.021		0.076	0.405	0.187	0.011	0.027	0.429	0.128	0.329	0.390	0.010	0.014	0.724	0.074	0.236	0.312	0.011	0.042	0.269
1995	0.023		0.082	0.427	0.191	0.005	0.012	0.432	0.107	0.392	0.272	0.031	0.043	0.732	0.092	0.299	0.309	0.015	0.067	0.230
1996	0.024		0.084	0.442	0.190	0.002	0.011	0.227	0.160	0.371	0.431	0.008	0.017	0.463	0.106	0.314	0.339	0.031	0.163	0.189
1997	0.022	0.011	0.056	0.437	0.129	0.012	0.016	0.735	0.120	0.362	0.331	0.019	0.039	0.495	0.081	0.307	0.263	0.024	0.114	0.214
1998	0.023		0.054	0.392	0.137	0.003	0.021	0.159	0.121	0.432	0.280	0.012	0.037	0.312	0.076	0.399	0.191	0.050	0.209	0.237
1999	0.021		0.037	0.345	0.108	0.001	0.009	0.154	0.090	0.488	0.184	0.011	0.005	2.115	0.071	0.398	0.178	0.007	0.050	0.144
2000	0.024		0.022	0.292	0.077	0.012	0.026	0.458	0.064	0.537	0.119	0.040	0.015	2.698	0.039	0.297	0.131	0.109	0.537	0.203
2001	0.022		0.021	0.217	0.098	0.001	0.009	0.112	0.133	0.172	0.775	0.009	0.023	0.379	0.055	0.351	0.158	0.006	0.047	0.118
2002	0.021		0.020	0.258	0.078	0.005	0.023	0.202	0.123	0.191	0.644	0.004	0.048	0.081	0.035	0.294	0.117	0.007	0.207	0.036
2003	0.023		0.011	0.179	0.061	0.001	0.009	0.063	0.060	0.144	0.415	0.001	0.011	0.093	0.016	0.224	0.071	0.003	0.049	0.061
2004	0.023		0.005	0.140	0.034	0.001	0.012	0.064	0.056	0.203	0.277	0.003	0.019	0.158	0.015	0.297	0.052	0.012	0.269	0.043
2005	0.023		0.005	0.181	0.025	0.000	0.010	0.049	0.050	0.243	0.205	0.003	0.002	1.333	0.006	0.173	0.037	0.002	0.090	0.027
2006	0.022	0.013	0.006	0.313	0.019	0.003	0.026	0.116	0.042	0.303	0.139	0.013	0.016	0.819	0.008	0.217	0.036	0.004	0.237	0.018
2007	0.021		0.021	0.629	0.034	0.001	0.038	0.028	0.052	0.172	0.305	0.003	0.009	0.293	0.006	0.155	0.038	0.001	0.044	0.021
2008	0.023		0.012	0.419	0.030	0.000	0.005	0.026	0.037	0.137	0.269	0.002	0.011	0.194	0.006	0.167	0.038	0.001	0.027	0.021
2009	0.023		0.011	0.461	0.023	0.001	0.022	0.066	0.037	0.182	0.205	0.002	0.002	0.820	0.005	0.100	0.049	0.003	0.166	0.020
2010	0.024		0.011	0.377	0.030	0.001	0.012	0.065	0.030	0.171	0.177	0.007	0.023	0.305	0.005	0.129	0.040	0.006	0.151	0.038
2011			0.019	0.586	0.032	0.001	0.030	0.020	0.040	0.123	0.325	0.001	0.013	0.058	0.004	0.053	0.083	0.001	0.071	0.010
2012	0.027		0.014	0.585	0.024	0.001	0.023	0.023	0.035	0.120	0.289	0.000	0.005	0.098	0.005	0.064	0.084	0.004	0.099	0.041
2013		0.013	0.012	0.601	0.019	0.002	0.064	0.032	0.037	0.171	0.214	0.002	0.011	0.152	0.006	0.055	0.103	0.005	0.169	0.029

Continue…..

Group num.	Saithe	Saithe	Saithe	Gurnards	Gurnards	Gurnards	Monkfish	Monkfish	Monkfish	Flatfish	Flatfish	Flatfish	Rays	Rays	Rays	Sharks	Sharks	Sharks	Large demersals	Large demersals
Type	4	6	0	4	6	0	4	6	0	4	6	0	4	6	0	4	6	0	4	6
1985	0.450	0.227	0.505	0.020	0.002	0.121	0.378	0.039	0.103	0.115	0.075	0.658	0.354	0.041	0.116	0.505	0.122	0.242	0.263	0.272
1986		0.302		0.026	0.002	0.072	0.658	0.030	0.046	0.137	0.064	0.465	0.260	0.035	0.133	0.479	0.082	0.170	0.672	0.233
1987	0.293	0.276	0.940	0.027	0.002	0.078	0.895	0.038	0.042	0.317	0.079	0.250	0.355	0.038	0.108	0.561	0.089	0.159	0.338	0.218
1988	0.096	0.273	2.855	0.045	0.002	0.051	0.695	0.056	0.081	0.132	0.091	0.693	0.990	0.043	0.043	0.737	0.074	0.100	0.633	0.228
1989	0.529	0.203	0.384	0.041	0.002	0.044	1.479	0.053	0.036	0.389	0.083	0.213	1.983	0.052	0.026	0.629	0.060	0.096	1.096	0.251
1990	0.201	0.162	0.805	0.021	0.002	0.076	0.657	0.052	0.079	0.294	0.080	0.271	0.210	0.037	0.175	0.361	0.051	0.142	0.621	0.193
1991	0.292	0.159	0.546	0.018	0.001	0.067	0.719	0.047	0.066	0.253	0.074	0.294	0.293	0.036	0.122	0.446	0.063	0.142	0.331	0.145
1992	0.105	0.099	0.944	0.007	0.001	0.118	0.439	0.052	0.118	0.131	0.067	0.514	0.069	0.033	0.475	0.524	0.060	0.115	0.098	0.121
1993	0.075	0.132	1.762	0.008	0.001	0.126	0.479	0.053	0.110	0.079	0.062	0.785	0.086	0.029	0.341	0.379	0.062	0.163	0.086	0.121
1994	0.314	0.117	0.372	0.005	0.001	0.133	0.298	0.051	0.170	0.086	0.060	0.698	0.075	0.026	0.343	0.305	0.053	0.173	0.092	0.112
1995	0.327	0.098	0.298	0.004	0.001	0.207	0.304	0.061	0.200	0.073	0.068	0.922	0.079	0.036	0.454	0.431	0.049	0.114	0.128	0.160
1996	0.077	0.086	1.120	0.004	0.001	0.144	0.322	0.061	0.189	0.082	0.060	0.730	0.221	0.044	0.199	0.413	0.042	0.101	0.127	0.128
1997	0.286	0.075	0.263	0.012	0.001	0.049	0.485	0.050	0.104	0.134	0.055	0.408	0.173	0.035	0.200	0.293	0.031	0.106	0.080	0.116
1998	0.023	0.066	2.945	0.005	0.001	0.137	0.217	0.042	0.195	0.044	0.048	1.084	0.115	0.030	0.256	0.112	0.024	0.211	0.052	0.141
1999	0.016	0.028	1.813	0.001	0.000	0.255	0.297	0.040	0.134	0.036	0.040	1.116	0.055	0.023	0.422	0.079	0.021	0.271	0.079	0.138
2000	0.114	0.060	0.528	0.001	0.000	0.390	0.237	0.030	0.127	0.028	0.034	1.183	0.055	0.023	0.427	0.408	0.055	0.136	0.013	0.164
2001	0.041	0.078	1.923	0.004	0.001	0.157	0.294	0.024	0.083	0.034	0.037	1.075	0.088	0.020	0.226	0.322	0.054	0.169	0.010	0.105
2002	0.031	0.051	1.628	0.003	0.001	0.285	0.129	0.021	0.164	0.019	0.030	1.602	0.034	0.020	0.589	0.108	0.035	0.322	0.011	0.094
2003	0.029	0.140	4.875	0.002	0.001	0.269	0.108	0.023	0.217	0.059	0.071	1.194	0.046	0.023	0.489	0.148	0.032	0.215	0.015	0.162
2004	0.032	0.052	1.600	0.001	0.000	0.376	0.083	0.023	0.278	0.066	0.050	0.755	0.051	0.020	0.387	0.160	0.029	0.180	0.012	0.128
2005	0.123	0.154	1.251	0.002	0.000	0.214	0.221	0.028	0.129	0.025	0.017	0.667	0.033	0.015	0.453	0.072	0.017	0.241	0.008	0.166
2006	0.103	0.171	1.663	0.001	0.000	0.355	0.111	0.026	0.238	0.015	0.011	0.758	0.017	0.007	0.401	0.026	0.010	0.380	0.005	0.135
2007	0.034	0.094	2.802	0.001	0.000	0.468	0.142	0.031	0.221	0.024	0.028	1.180	0.010	0.007	0.655	0.018	0.007	0.375	0.007	0.143
2008	0.013	0.127	6.603	0.001	0.000	0.404	0.267	0.032	0.119	0.006	0.005	0.837	0.011	0.006	0.543	0.009	0.003	0.296	0.010	0.144
2009	0.052	0.079	1.514	0.004	0.002	0.489	0.400	0.028	0.069	0.005	0.006	1.183	0.003	0.002	0.737	0.012	0.004	0.347	0.012	0.183
2010	0.034	0.089	2.594	0.003	0.002	0.598	0.238	0.029	0.122	0.009	0.008	0.900	0.005	0.004	0.713	0.002	0.001	0.548	0.018	0.187
2011	0.034	0.127	3.749	0.005	0.002	0.276	0.294	0.025	0.084	0.010	0.007	0.650	0.012	0.004	0.345	0.005	0.001	0.221	0.036	0.249
2012	0.107	0.167	1.552	0.012	0.002	0.192	0.168	0.026	0.153	0.022	0.020	0.895	0.010	0.004	0.415	0.009	0.003	0.301	0.029	0.223
2013	0.074	0.143	1.930	0.010	0.002	0.188	0.144	0.023	0.160	0.017	0.014	0.813	0.015	0.004	0.282	0.041	0.008	0.189	0.042	0.235

Continue…..

Group num.	Large demersals	Bentho-pelagic fish	Bentho-pelagic fish	Bentho-pelagic fish	Mack erel	Mackerel	Mackerel	Horse Mackerel	Horse Mackerel	Horse Mackerel	Blue Whiting	Blue Whiting	Blue Whiting	Herring	Herring	Herring	Norway pout	Norway pout	Norway pout	Poor cod
Type	0	4	6	0	4	6	0	4	6	0	4	6	0	4	6	0	4	6	0	0
1985	1.034	0.008	0.005	0.585	0.118	1.017	8.638	0.045	0.300	6.621	0.443	0.876	1.978	0.060	0.358	5.943	0.008	0.010	1.290	0.071
1986	0.347	0.415	0.021	0.051	0.126	0.997	7.926	0.027	0.185	6.959	0.659	1.212	1.839	0.119	0.674	5.676	0.050	0.053	1.071	0.027
1987	0.647	0.036	0.009	0.254	0.142	1.085	7.623	0.047	0.320	6.860	0.396	0.622	1.570	0.050	0.392	7.899	0.195	0.348	1.780	0.025
1988	0.359	0.898	0.073	0.081	0.144	1.127	7.832	0.062	0.417	6.728	0.356	0.481	1.350	0.053	0.331	6.221	0.044	0.061	1.396	0.095
1989	0.229	8.707	0.188	0.022	0.133	0.970	7.317	0.049	0.317	6.406	0.464	0.630	1.360	0.103	0.581	5.629	0.354	0.256	0.724	0.018
1990	0.310	1.281	0.063	0.049	0.144	1.037	7.201	0.034	0.189	5.575	0.438	0.600	1.370	0.165	0.802	4.846	0.016	0.030	1.890	0.038
1991	0.438	0.244	0.044	0.180	0.156	1.119	7.151	0.059	0.313	5.274	0.187	0.334	1.783	0.160	0.620	3.874	0.028	0.040	1.415	0.095
1992	1.229	0.048	0.051	1.079	0.201	1.260	6.266	0.087	0.372	4.286	0.251	0.522	2.076	0.169	0.554	3.279	0.017	0.047	2.760	0.285
1993	1.402	0.030	0.011	0.365	0.238	1.365	5.733	0.120	0.489	4.072	0.289	0.579	2.004	0.186	0.621	3.335	0.018	0.067	3.681	0.211
1994	1.215	0.695	0.071	0.102	0.271	1.356	4.999	0.171	0.632	3.693	0.340	0.645	1.901	0.170	0.530	3.114	0.028	0.129	4.552	0.134
1995	1.254	0.143	0.046	0.322	0.254	1.252	4.924	0.238	0.759	3.187	0.361	0.684	1.897	0.202	0.522	2.584	0.051	0.222	4.373	0.089
1996	1.007	0.077	0.075	0.975	0.204	0.933	4.569	0.240	0.739	3.080	0.360	0.765	2.128	0.175	0.544	3.110	0.012	0.057	4.810	0.101
1997	1.452	0.644	0.068	0.106	0.204	0.949	4.647	0.130	0.365	2.807	0.343	1.079	3.143	0.176	0.570	3.242	0.021	0.087	4.078	0.112
1998	2.728	0.282	0.057	0.202	0.244	1.103	4.528	0.148	0.319	2.148	0.392	1.566	3.999	0.233	0.657	2.819	0.006	0.065	10.729	0.365
1999	1.748	0.217	0.079	0.363	0.206	1.060	5.136	0.189	0.367	1.940	0.278	1.179	4.239	0.225	0.508	2.256	0.010	0.042	4.249	0.302
2000	12.290	0.156	0.109	0.699	0.241	1.223	5.069	0.107	0.188	1.750	0.240	1.021	4.251	0.125	0.347	2.781	0.003	0.018	6.191	0.238
2001	10.512	0.222	0.132	0.595	0.265	1.221	4.610	0.152	0.224	1.474	0.234	1.223	5.223	0.120	0.358	2.995	0.005	0.029	6.106	0.296
2002	8.256	0.025	0.108	4.368	0.265	1.277	4.827	0.074	0.129	1.736	0.120	0.696	5.802	0.135	0.462	3.421	0.006	0.044	6.883	0.274
2003	10.566	0.023	0.079	3.375	0.208	1.125	5.410	0.085	0.211	2.482	0.086	0.603	6.988	0.136	0.407	2.997	0.018	0.059	3.283	0.382
2004	10.920	0.005	0.050	10.331	0.201	1.094	5.437	0.070	0.199	2.855	0.173	1.069	6.179	0.145	0.357	2.466	0.008	0.021	2.751	0.460
2005	20.861	0.039	0.054	1.382	0.179	0.910	5.090	0.068	0.200	2.950	0.248	1.247	5.019	0.124	0.276	2.227	0.000	0.000	0.833	0.455
2006	27.085	0.003	0.010	3.166	0.143	0.797	5.591	0.057	0.143	2.518	0.347	1.611	4.642	0.189	0.423	2.237	0.000	0.000	1.004	0.554
2007	19.285	0.003	0.043	12.212	0.168	1.011	6.033	0.110	0.239	2.180	0.386	1.333	3.456	0.229	0.431	1.879	0.000	0.000	3.588	1.154
2008	13.873	0.002	0.028	16.718	0.139	1.003	7.208	0.102	0.235	2.304	0.496	1.298	2.618	0.158	0.268	1.691	0.000	0.000	1.950	0.712
2009	15.331	0.001	0.001	2.040	0.169	1.325	7.847	0.066	0.162	2.453	0.427	0.885	2.071	0.159	0.272	1.708	0.000	0.000	5.408	0.890
2010	10.205	0.009	0.027	3.108	0.125	1.053	8.416	0.094	0.206	2.179	0.342	0.805	2.354	0.151	0.273	1.802	0.000	0.000	10.729	0.408
2011	6.869	0.010	0.015	1.478	0.233	2.210	9.480	0.183	0.357	1.950	0.042	0.124	2.951	0.141	0.224	1.592	0.000	0.000	7.758	0.197
2012	7.659	0.006	0.022	3.722	0.124	1.132	9.108	0.255	0.409	1.604	0.129	0.451	3.498	0.153	0.228	1.484	0.000	0.000	3.467	0.426
2013	5.616	0.009	0.019	2.038	0.126	1.157	9.209	0.303	0.393	1.299	0.110	0.444	4.024	0.215	0.244	1.137	0.000	0.000	2.149	0.277

Continue….

Group num.	Sandeel	Sandeel	Sandeel	Sandeel	Sprat	Sprat	Sprat	Norway lobster	Norway lobster	Norway lobster	Norway lobster	Lobster	Edible crab	Velvet crab	Crustaceans	Cephalopod	Large zooplankton	Small zooplankton	Scallops	Epifauna	Phytoplankton
Type	4	6	0	-6	4	6	0	4	6	0	-6	-6	-6	-6	-6	-6	0	0	-6	-6	0
1985	0.129	0.169	1.314		0.100	0.032	0.322				0.114	0.006	0.044	0.004	0.000	0.000	0.072	0.995	0.060	0.000	0.242
1986	0.102	0.222	2.174		0.017	0.008	0.468				0.103	0.005	0.041	0.006	0.000	0.000	0.072	0.893	0.053	0.000	0.268
1987	0.041	0.132	3.213		0.034	0.008	0.226				0.103	0.006	0.071	0.011	0.000	0.001	0.089	0.789	0.063	0.001	0.273
1988	0.120	0.222	1.853		0.021	0.040	1.930				0.116	0.007	0.098	0.012	0.000	0.000	0.043	0.665	0.045	0.000	0.268
1989	0.130	0.171	1.309		0.060	0.012	0.197				0.100	0.008	0.085	0.011	0.002	0.003	0.066	0.711	0.057	0.000	0.296
1990	0.094	0.150	1.599		0.090	0.015	0.162				0.092	0.007	0.071	0.013	0.002	0.002	0.080	0.739	0.039	0.000	0.251
1991	0.054	0.078	1.443		0.052	0.015	0.296				0.095	0.007	0.089	0.014	0.002	0.001	0.072	0.879	0.038	0.000	0.253
1992	0.011	0.045	4.137		0.090	0.018	0.196				0.099	0.008	0.092	0.019	0.004	0.001	0.045	0.759	0.045	0.001	0.257
1993	0.014	0.057	4.178		0.061	0.042	0.691				0.103	0.006	0.084	0.018	0.005	0.001	0.060	0.865	0.067	0.003	0.251
1994				0.097	0.072	0.014	0.194	0.344	0.103	0.300		0.005	0.106	0.018	0.005	0.002	0.076	0.881	0.070	0.003	0.268
1995				0.065	0.036	0.040	1.110	0.354	0.117	0.331		0.006	0.102	0.027	0.003	0.003	0.115	0.844	0.062	0.001	0.286
1996				0.121	0.019	0.024	1.242	0.162	0.101	0.625		0.004	0.080	0.008	0.002	0.002	0.052	0.690	0.063	0.006	0.288
1997				0.115	0.035	0.063	1.811	0.207	0.102	0.494		0.006	0.109	0.018	0.002	0.002	0.080	0.673	0.077	0.006	0.298
1998				0.048	0.116	0.033	0.283	0.115	0.102	0.885		0.006	0.110	0.015	0.003	0.001	0.082	0.680	0.090	0.001	0.315
1999				0.024	0.044	0.100	2.254	0.138	0.107	0.770		0.004	0.107	0.014	0.002	0.000	0.111	0.831	0.058	0.003	0.288
2000				0.052	0.029	0.070	2.419	0.081	0.102	1.264		0.003	0.116	0.009	0.001	0.000	0.107	0.805	0.109	0.014	0.329
2001				0.003	0.005	0.013	2.742	0.087	0.106	1.220		0.003	0.142	0.012	0.001	0.000	0.066	0.855	0.124	0.005	0.319
2002				0.006	0.081	0.036	0.448	0.075	0.100	1.333		0.003	0.140	0.012	0.001	0.000	0.117	0.955	0.196	0.003	0.331
2003				0.000	0.055	0.041	0.748	0.073	0.111	1.517		0.003	0.144	0.008	0.004	0.001	0.084	0.911	0.121	0.007	0.311
2004				0.005	0.003	0.014	4.920	0.065	0.109	1.678		0.005	0.170	0.012	0.003	0.001	0.090	0.875	0.112	0.006	0.268
2005				0.000	0.027	0.013	0.464	0.069	0.107	1.549		0.003	0.098	0.004	0.002	0.000	0.090	0.815	0.068	0.001	0.295
2006				0.000	0.008	0.005	0.645	0.098	0.138	1.413		0.007	0.168	0.001	0.001	0.001	0.094	0.796	0.085	0.005	0.220
2007				0.000	0.005	0.003	0.603	0.197	0.179	0.908		0.007	0.242	0.000	0.002	0.001	0.071	0.748	0.054	0.003	0.280
2008				0.000	0.016	0.008	0.506	0.131	0.156	1.192		0.007	0.142	0.019	0.002	0.003	0.083	0.744	0.087	0.002	0.223
2009				0.000	0.004	0.002	0.533	0.107	0.131	1.224		0.006	0.129	0.019	0.001	0.003	0.065	0.707	0.097	0.002	0.321
2010				0.000	0.015	0.013	0.879	0.081	0.117	1.446		0.007	0.155	0.019	0.003	0.004	0.120	0.912	0.113	0.002	0.271
2011				0.000	0.025	0.016	0.619	0.090	0.123	1.363		0.006	0.161	0.015	0.002	0.001	0.083	0.820	0.140	0.003	0.322
2012				0.000	0.271	0.032	0.117	0.198	0.141	0.715		0.006	0.139	0.016	0.004	0.001	0.073	0.828	0.198	0.003	0.285
2013				0.000	0.010	0.022	2.264	0.079	0.121	1.538		0.004	0.135	0.011	0.003	0.001	0.103	0.778	0.109	0.003	0.294

Supplementary Table S7. Fitted vulnerabilities. The stepwise fitting procedure estimated 33 vulnerabilities of prey to these predators: values greater than 2 showed sensitive predator top-down controls on their preys whilst values between 1 and 2 indicated bottom-up controlled tropic relationships.

Group num.	vulnerability of prey to predator	Group num.	vulnerability of prey to predator
1-Grey seals	2.3	20-Horse Mackerel	1.05
2-Harbour seals	2	21-Blue Whiting	1.88
3-Cetaceans	>1,000	22-Other pelagics	1
4 Seabirds	1	23-Herring	1.84
5 Cod mature	2.8	24-Norway pout	2
6-Cod juv.	1.11	25-Poor cod	1.7
7-Haddock mature	1	26-Sandeel	1
8-Haddock juv	>1,000	27-Sprat	16.03
9-Whiting mature	2.6	28-Norway lobster	1.52
10-Whiting juv.	>1,000	29-Lobster	2
11-Saithe	12.3	30-Edible crab	1
12-Gurnards	2	31-Velvet crab	1
13- Monkfish	1	32-Crustaceans	1
14-Flatfish	1.48	33 Cephalopod	1
15- Rays	1	34-Large zooplankton	1
16-Sharks	1.23	35 Small zooplankton	1
17-Large demersals	1	36-Infauna	2
18- Benthopelagic fish	1.25	37-Scallops	1
19-Mackerel	1	38-Epifauna	>1,000

Supplementary Table S8. Maximum and minimum temperatures and preference quintiles of species within each functional group. Averaged optimum temperatures, and left-right standard deviations (SD) for each functional group with data sources are also shown.

Group num.	EwE groups	Species common names	*Species*	Minimum temperature	10th percentile	90th percentile	Maximum temperature	Optimum temperature	Optimum EwE group temperature (weighted by composition)	SD left	SD right	Data source
1	Grey seals	Grey seals	*Halichoerus grypus*	2	5	10	15	7.5	7.5	2	2.5	Aquamaps[26]
2	Harbour seals	Harbour seals	*Phoca vitulina*	-1.75	5	10	15	7.5	7.5	3.2	2.5	Aquamaps[26]
3	Cetaceans	Minke whale	*Balaenoptera acutorostrata*	-2	-1.61	15.67	29.31	7.03	13	3.4	3.5	Aquamaps[26]
		Harbour Porpoises	*Phocoena phocoena*	0	5	15	20	10				
		common dolphins	*Delphinus delphis*	5	12	27.42	31.62	19.71				
		common dolphins	*Tursiops truncatus*	5	12	28.16	30	20.08				
		common dolphins	*Lagenorhynchus albirostris*	-0.49	3.22	10	15	6.61				
4	Seabirds	little auks	*Alle alle*	-2	-1.59	10.67	22.34	4.54	12.9	4.2	4.7	Aquamaps[26], SeaLifeBase[27]
		petrels	*Calonectris diomedea*	6.34	10.12	26.05	30.25	18.085				
		petrels	*Oceanodroma leucorhoa*	-0.6	3.98	27.23	31.43	15.605				
		petrels	*Puffinus puffinus*	3.27	8.64	21.19	27.47	14.915				
		pelecaniformes	*Phalacrocorax carbo*	-1.82	6.14	19.5	28.97	12.82				
		pelecaniformes	*Phalacrocorax aristotelis*	3.63	7.35	17.98	21.49	12.665				
		gulls	*Larus atricilla*	-1.59	10.67	26.72	30.92	18.695				
		gulls	*Larus argentatus*	-1.76	2.54	23.84	28.47	13.19				
		guillemot	*Uria aalge*	-1.73	2.06	12.31	21.1	7.185				
		razorbill	*Alca torda*	-1.61	3.54	12.66	21.93	8.1				
		Atlantic puffin	*Fratercula artica*	-1.79	0.52	12.54	21.93	6.53				
		Manx shearwater	*Puffinus puffinus*	-0.61	6.14	19.39	28.38	12.765				
5	Cod		*Gadus morhua*	-1.66	2.54	12.29	15	7.415	6.3	2.5	2.35	FishBase[28],optimum temperature[29], tolerances[26] and adjustments for juveniles[30]
7	Haddock	Haddock	*Melanogrammus aeglefinus*	0.17	4.69	12.01	22.83	8.35	7	2.3	4	FishBase[28],Aquamaps[26]and adjustment for juveniles[30]
9	Whiting	Whiting	*Merlangius merlangus*	6.14	9.31	12.5	18.6	10.905	9.5	1.2	3	FishBase[28],Aquamaps[26]and adjustment for juveniles[30]
11	Saithe	Pollock	*Pollachius pollachius*	6.33	9.6	12.53	19.3	11.065	9	3	4.3	Aquamaps[26]
		Saithe	*Pollachius virens*	-0.4	5.41	12.5	22.1	8.955				

Continue….

Group num.	EwE groups	Species common names	*Species*	Minimum temperature	10th percentile	90th percentile	Maximum temperature	Optimum temperature	Optimum EwE group temperature (weighted by composition)	SD left	SD right	Data source
12	Gurnard	Grey gurnard	*Eutrigla gurnardus*	5.77	9.65	16.04	22.07	12.845	14.1	3.1	2.6	Aquamaps[26]
		Piper gurnard	*Trigla lyra*	3.82	13.98	27.89	32.09	20.935				
		Red gurnard	*Aspitrigla cuculus*	6.03	9.77	18.7	22.5	14.235				
		Streaked gurnard	*Trigloporus lastoviza*	6.7	11.19	27.66	31.86	19.425				
		Tub gurnard	*Trigla lucerna*	5.86	9.67	18.95	27.39	14.31				
13	Monkfish	Anglerfish	*Lophius piscatorius*	2.47	9.41	13.43	16.28	11.42	11.42	3.2	1.5	Aquamaps[26]
		Black bellied angler	*Lophius budegassa*	2.47	9.41	13.43	16.28	11.42				
14	Flatfish	Atlantic halibut	*Hippoglossus hippoglossus*	-0.99	0	9.92	16.6	4.96	12.3	2.6	3	Aquamaps[26]
		Atlantic sole	*Pegusa lascaris*	10.12	11.45	27.69	31.89	19.57				
		Brill	*Scophthalmus rhombus*	1.31	9.3	19.1	25.71	14.2				
		Common European sole	*Solea vulgaris*	6.965	9.76	18.98	25	14.37				
		Common topknot	*Zeugopterus punctatus*	6.95	9.72	12.48	17.4	11.1				
		Dab	*Limanda limanda*	2.98	9.04	12.49	15.62	10.765				
		European flounder	*Platichthys flesus*	0	8.27	12.48	23.33	10.375				
		European plaice	*Pleuronectes platessa*	-1.77	8.8	13.4	18	11.1				
		Fourspot megrim	*Lepidorhombus boscii*	4.3	12.07	19.23	26.27	15.65				
		Grohmann's scaldfish	*Arnoglossus thori*	14.25	18.27	25.09	29.29	21.68				
		Imperial scaldfish	*Arnoglossus imperialis*	6.17	12.81	27.92	28.42	20.365				
		Lemon sole	*Microstomus kitt*	2.03	9.62	12.83	15.73	11.225				
		Long rough dab	*Hippoglossoides platessoides*	-1.66	1.08	11.3	18.2	6.19				
		Megrim	*Lepidorhombus whiffiagonis*	5.28	9.65	14.92	21.17	12.285				
		Norwegian topknot	*Phrynorhombus norvegicus*	5.91	9.68	13.26	15.88	11.47				
		Scaldfish	*Arnoglossus laterna*	2.27	9.81	19.66	28.06	14.735				
		Solenette	*Buglossidium luteum*	3.5	8.4	18.27	26.4	13.335				
		Thickback sole	*Microchirus variegatus*	2.68	11.45	19.84	27.88	15.645				
		Turbot	*Psetta maxima*	6.7	8.93	12.49	21.91	10.71				
		Witch flounder	*Glyptocephalus cynoglossus*	-1.34	1.91	11.43	19.19	6.67				

Continue….

Group num.	EwE groups	Species common names	*Species*	Minimum temperature	10th percentile	90th percentile	Maximum temperature	Optimum temperature	Optimum EwE group temperature (weighted by composition)	SD left	SD right	Data source
15	Rays and skates	Blonde ray	*Raja brachyura*	1.24	10.95	19.96	29.2	15.455	12	3.2	3.6	Aquamaps[26]
		Blue skate	*Dipturus batis*	2.52	7.08	13.07	16.98	10.075				
		Bottlenosed skate	*Rostroraja alba*	-1.985	3.24	21.71	25.73	12.475				
		Cuckoo ray	*Leucoraja circularis*	4.69	10.26	19.19	23.97	14.725				
		Cuckoo ray	*Raja naevus*	5.28	9.77	16.75	22.2	13.26				
		Homelyn Ray	*Raja montagui*	1.38	12	20.64	29.62	16.32				
		Shagreen ray	*Leucoraja fullonica*	4.035	9.63	18.5	27.22	14.065				
		Starry skate	*Amblyraja radiata*	-1.3	-1	5	14	2				
		Thornback Ray	*Raja clavata*	6.45	10.71	27.3	31.5	19.005				
16	Sharks	Blackmouth catshark	*Galeus melastomus*	-1.03	9.8	20.62	24.51	15.21	16	5.5	3.6	Aquamaps[26]
		Nursehound	*Scyliorhinus stellaris*	-0.73	10.71	21.14	25.5	15.925				
		Picked dogfish	*Squalus acanthias*	-1.66	7.42	18.94	28.9	13.18				
		Small-spotted catshark	*Scyliorhinus canicula*	-1.67	10.61	27.56	31.76	19.085				
		Smooth-hound	*Mustelus mustelus*	9.68	10.51	27.66	31.86	19.085				
		Starry smooth-hound	*Mustelus asterias*	4.69	9.75	19.95	22.5	14.85				
		Tope	*Galeorhinus galeus*	3	5.5	15	26	10.25				
17	Large demersal	Blue-Mouth	*Helicolenus dactylopterus*	0.55	9.83	27.09	31.29	18.46	14	3.2	3.1	Aquamaps[26]
		Sea trout	*Salmo trutta*	1.18	6.9	14	21.17	10.45				
			Salmo salar	-0.82	2.17	11.57	20.23	6.87				
		Catfish	*Anarhichas lupus*	-0.77	1.08	9.91	17.96	5.495				
		Common eel	*Anguilla anguilla*	3.8	8.98	23.34	27.5	16.16				
		Conger eel	*Conger conger*	2.02	2.52	13.3	24.935	7.91				
		Cusk	*Brosme brosme*	0	2.74	9	10	5.87				
		Greater Forkbeard	*Phycis blennoides*	0.61	9.72	19.33	29.13	14.525				
		Hake	*Merluccius merluccius*	5.01	11.07	22.07	28.93	16.57				
		John Dory	*Zeus faber*	-1.74	9.94	26.32	30.52	18.13				

Continue...

Group num.	EwE groups	Species common names	*Species*	Minimum temperature	10th percentile	90th percentile	Maximum temperature	Optimum temperature	Optimum EwE group temperature (weighted by composition)	SD left	SD right	Data source
17	Large demersal	Ling	*Molva molva*	0.55	9.62	12.9	19.35	11.26	14	3.2	3.1	Aquamaps[26]
		Lumpsucker	*Cyclopterus lumpus*	-1.66	2.47	10.87	22.95	6.67				
		Norway haddock	*Sebastes viviparus*	1.61	9.19	10.68	12.01	9.935				
		Ocean perch	*Sebastes marinus (norvegicus?)*	-1.66	0.12	9.68	18.34	4.9				
		Red bandfish	*Cepola rubescens (macrophthalma)*	3.38	12.01	21.14	28.54	16.575				
		Red sea bream	*Pagellus bogaraveo*	6.1	10.34	20.2	27.11	15.27				
18	Benthopelagic fish	Alaskan Stickleback	*Gasterosteus aculeatus*	0.4	2.46	12.07	14.9	7.265	15	5	2	Aquamaps[26]
		Atlantic argentine	*Argentina silus*	-1.66	5.41	12.9	16.07	9.155				
		Atlantic warbonnet	*Chirolophis ascanii*	0.67	7.95	12.17	13.05	10.06				
		Ballan wrasse	*Labrus bergylta*	6.7	9.68	12.9	21.25	11.29				
		Bib	*Trisopterus luscus*	6.85	9.77	14.78	20.44	12.275				
		Boarfish	*Capros aper*	1.72	10.53	20.09	28.92	15.31				
		Bridled triggerfish	*Balistes carolinensis*	-0.74	2.29	24.34	28.02	13.315				
		Bull rout	*Myoxocephalus scorpius*	-1.78	-0.38	10.92	19.16	5.27				
		Butterfish	*Pholis gunnellus*	-1.66	6.05	12.01	15.33	9.03				
		Common dragonet	*Callionymus lyra*	6.14	16	20	24.77	18				
		Common goby	*Pomatoschistus microps*	6.34	9.05	15.78	19.15	12.415				
		Crystal goby	*Crystallogobius linearis*	5.91	9.5	19.06	20.33	14.28				
		Cuckoo wrasse	*Labrus mixtus*	5.91	9.83	19.1	24.73	14.465				
		Echiodon drummondii	*Echiodon drummondii*	5.99	9.61	12.6	16.07	11.105				
		Fivebearded rockling	*Ciliata mustela*	1.05	8	11.67	13.94	9.835				
		Fourbeard rockling	*Enchelyopus cimbrius*	-1.66	4.6	13.18	26.67	8.89				
		Freckled goby	*Pomatoschistus minutus*	5.91	9.12	14.01	21.17	11.565				
		Fries' goby	*Lesueurigobius friesii*	0.08	9.68	19.03	28.16	14.355				
		Goldsinny wrasse	*Ctenolabrus rupestris*	6.67	9.65	15.47	22.07	12.56				
		Great Pipefish	*Syngnathus acus*	8	11	24.21	27	17.605				

Continue….

Group num.	EwE groups	Species common names	*Species*	Minimum temperature	10th percentile	90th percentile	Maximum temperature	Optimum temperature	Optimum EwE group temperature (weighted by composition)	SD left	SD right	Data source
18	Benthopelagic fish	Hook-nose	*Agonus cataphractus*	2.99	9.3	12.13	14.92	10.715	15	5	2	Aquamaps[26]
		Lesse forkbeard	*Raniceps raninus*	7.43	9.5	12.38	14.92	10.94				
		Lesser weever	*Echiichthys vipera*	7.43	10.04	17.64	22.07	13.84				
		Longspine snipefish	*Macroramphosus scolopax*	0.67	13.78	24.99	29.69	19.385				
		Longspined sea scorpion	*Taurulus bubalis*	5.91	8.92	12.5	19.35	10.71				
		Moustache sculpin	*Triglops murrayi*	-1.66	0.96	9.89	16.15	5.425				
		Northern rockling	*Ciliata septentrionalis*	-0.73	3.03	11.43	18.575	7.23				
		Norway bullhead	*Taurulus lilljeborgi*	5.91	8.92	12.5	19.35	10.71				
		Ocean Pipefish	*Entelurus aequoraeus*	7.04	9.72	13.97	18.7	11.845				
		Red mullet	*Mullus surmuletus*	1.24	9.72	19.57	24.63	14.645				
		Reticulated dragonet	*Callionymus reticulatus*	7.8	9.77	12.48	19.26	11.125				
		Silver smelt	*Argentina sphyraena*	4.51	9.63	18.43	21.21	14.03				
		Silvery pout	*Gadiculus argenteus*	-0.945	9.62	19.35	20.18	14.485				
		Snake blenny	*Lumpenus lumpretaeformis*	-1.74	0.61	9.5	10.9	5.055				
		Spotted dragonet	*Callionymus maculatus*	5.52	9.65	16.07	22.07	12.86				
		Striped seasnail	*Liparis liparis*	-1.66	6.6	11.92	14.97	9.26				
		Threebearded rockling	*Gaidropsarus vulgaris*	2.52	4.86	13.19	17.7	9.025				
		Tompot blenny	*Blennius gattorugine*	3.5	10.67	18.95	24.7	14.81				
		True gobies	*Gobiidae(Pomatoschistus microps)*	6.34	9.05	15.78	19.15	12.415				
		Viviporous blenny	*Zoarces viviparus*	5.19	7.31	10.79	13.09	9.05				
19	Mackerel	Atlantic mackerel	*Scomber scombrus*	3	7.5	18.38	27.15	12.94	12.94	3.2	4.5	Aquamaps[26]
20	Horse mackerel	Horse mackerel	*Trachurus trachurus*	3.93	9.65	19.25	27.38	14.45	14.45	3	4	Aquamaps[26]
21	Blue Whiting	Blue whiting	*Micromesistius poutassou*	0.61	8.24	19.03	28.37	13.635	13.6	4.3	4.5	Aquamaps[26]

Continue...

Group num.	EwE groups	Species common names	*Species*	Minimum temperature	10th percentile	90th percentile	Maximum temperature	Optimum temperature	Optimum EwE group temperature (weighted by composition)	SD left	SD right	Data source
22	Other pelagic	Alice shad	*Alosa alosa*	3.81	9.68	18.51	21.94	14.095	15	4	4	Aquamaps[26]
		European anchovy	*Engraulis encrasicolus*	4.13	11.57	27.89	32.09	19.73				
		Pearlside	*Maurolicus muelleri*	-1.26	9.1	20.63	28.33	14.865				
		True sardine	*Sardina pilchardus*	2.53	12	26.92	27.62	19.46				
		Twaite shad	*Alosa fallax*	2.27	9.3	18.33	27.76	13.815				
23	Herring	Herring	*Clupea harengus*	-1.34	-0.1	9.34	24.7	4.62	4.62	1.8	5	Aquamaps[26]
24	Norway pout	Norway pout	*Trisopterus esmarkii*	-1.66	9.57	12.52	13.72	11.045	11	4	1	Aquamaps[26]
25	Poor cod	Poor Cod	*Trisopterus minutus*	5.84	9.65	16.04	22.07	12.845	12.85	2.3	3	Aquamaps[26]
26	Sandeel	Corbin's sand eel	*Hyperoplus immaculatus*	8.09	10.19	13.18	15.18	11.685	11	2.5	1.3	Aquamaps[26]
		Greater sand eel	*Hyperoplus lanceolatus*	6.68	9.01	12	17.69	10.505				
		Sand eel	*Ammodytes marinus*	-1.66	9.44	11.33	12.5	10.385				
		Smoothed sandeel	*Gymnammodytes semisquamatus*	7.56	9.76	12.81	16.28	11.285				
27	Sprat	Sprat	*Sprattus sprattus*	3.185	7.78	18.27	27.89	13.025	13	3.2	4.5	Aquamaps[26]
28	Nephrops	Nephrops	*Nephrops norvegicus*	5.35	9.68	18.22	21.21	13.95	13.95	3	2.4	SeaLifeBase[27]
29	Lobster	Lobster	*Palinurus elephas*	-0.29	10.06	19.33	28.95	14.695	14.7	6	6	SeaLifeBase[27]
30	Edible crab	Edible crab	*Cancer pagurus*	7.04	9.75	12.81	20.15	11.28	11.28	1.5	2.9	SeaLifeBase[27]
31	Velvet crab	Velvet crab	*Necora puber*	4.06	11	19.12	20.15	15.06	15	2.8	1.8	SeaLifeBase[27]
32	Crustaceans	Blue crab	*Callinectes sapidus*	0.67	12.52	27.56	31.76	20.04	13.5	3.5	3.4	SeaLifeBase[27]
		Common prawn	*Palaemon serratus*	8.11	9.89	17.56	27.59	13.725				
		Common shrimp	*Crangon crangon*	6.19	9.65	13.4	22.07	11.525				
		Craylets, squat lobsters	*Munida gregaria*	6.38	9.6	12.83	16.74	11.215				
		Deep-sea red crab	*Chaceon affinis*	5.22	8.79	23.89	29.01	16.34				
		Green crab	*Carcinus maenas*	7.15	9.65	12.5	22.5	11.075				
		Northern prawn	*Pandalus montagui*	6.86	9.74	12.83	14.86	11.285				
		Spinous spider crab	*Maja squinado*	1.99	10.71	23.89	28.01	17.3				
		Portunus swimcrabs nei	*Portunus pelagicus*	12.24	17.37	28.6	32.8	22.985				

Continue...

Group num.	EwE groups	Species common names	*Species*	Minimum temperature	10th percentile	90th percentile	Maximum temperature	Optimum temperature	Optimum EwE group temperature (weighted by composition)	SD left	SD right	Data source
33	Cephalopod	Broadtail shortfin squid	*Illex coindetii*	0.67	12.37	27.67	31.87	20.02	14.9	6	5	Aquamaps[26]
		Common cuttlefish	*Sepia officinalis*	0.41	10.51	20.98	22.5	15.745				
		Common octopus	*Octopus vulgaris*	0.67	11.55	27.96	32.16	19.755				
		Common squids	*Alloteuthis subulata*	9.17	11.66	20.71	25.46	16.185				
		Cuttlefish,bobtail squids	*Sepiola atlantica*	5.91	9.65	21.27	28.5	15.46				
		European flying squid	*Todarodes sagittatus*	2.39	9.6	21.83	24.02	15.715				
		Inshore squids	*Doryteuthis (Loligo) ocula*	8.5	9.68	28.5	30	19.09				
		Northern shortfin squid	*Illex illecebrosus*	0.55	4.69	21.1	26.31	12.895				
34	Large zooplankton	Jellyfish	*Aurelia aurita*	-1.85	7.08	26.02	30.22	16.55	12.5	6	6	Aquamaps[26]for *Aurelia aurita*
		Jellyfish	*Cyanea lamarckii*	-	-	-	-	-				
		Jellyfish	*Cyanea capillata*	-	-	-	-	-				
		Hyperiid	*Themisto spp*	-	-	-	-	-				
		Hyperiid	*Phronima spp*	-	-	-	-	-				
		Chaetognaths	*Sagitta spp*	-	-	-	-	-				
35	Small zooplankton	Calanoida	*Calanus finmarchicus*	-	-	-	-	-	12.5	6	6	-
		Calanoida	*Calannus helgolandicus*	-	-	-	-	-				
		Calanoida	*Oithona similes*	-	-	-	-	-				
		meroplankton and tunicates < 5 mm		-	-	-	-	-				
36	Infauna	Sipunculida	*Golfingia sp*	-	-	-	-	-	14	5	5	SeaLifeBase[27]
		Bivalve	*Mysella bidentata*	-	-	-	-	-				
		Bivalve	*Lucinoma borealis*	0.67	2.39	25.33	29.53	13.86				
		Bivalve	*Hiatella arctica*	-1.79	5.91	23.28	28.68	14.595				
37	Scallops		*Aequipecten opercularis*	1.15	9.76	19.15	25.71	14.455	11.4	2.3	2.6	Aquamaps[26]
			Pecten maximus	7.35	9.83	14.03	20.35	11.93				

Continue...

Group num.	EwE groups	Species common names	*Species*	Minimum temperature	10th percentile	90th percentile	Maximum temperature	Optimum temperature	Optimum EwE group temperature (weighted by composition)	SD left	SD right	Data source
38	Epifauna	Gastropod	*Bittium reticulatum*	0.33	9.72	19.34	28.65	14.53	12	5	5	SeaLifeBase[27]
		Gastropod	*Gibbula cineraria*	2.43	10.31	18.95	24.59	14.63				
		Decapod	*Porcellana longicornis (used Liocarcinus depurator)*	9.5	10.95	16.5	20.15	13.725				
		Gastropod	*Rissoa parva*	-	-	-	-	-				
		Isopod	*Idotea spp.*	8.45	9.62	12.39	13.41	11.005				
		Gastropod	*Tricolia pullus*	9.19	9.98	12.01	13.15	10.995				
		Decapod	*Xantho spp (used Liocarcinus depurator)*	3.04	3.98	12.38	15.13	8.18				
		Bivalve	*Musculus discors (used Modiolula phaseolina)*	5.55	8.97	18.22	22.24	13.595				
		Sea stars	*Asterias rubens*	7.75	9.77	12.38	13.87	11.075				
		Urchins	*Psammechinus miliaris*	-	-	-	-	-				
		Crinoid	*Leptometra*	9.62	10.67	14.02	19.26	12.345				
39	Algae	Kelp species	Laminaria saccharina	-1.5283333	0.110234	11.65892	15.72809262	5.8847	8	4	2	SNH report[31]
			Laminaria digitata	-0.58249999	1.105254	11.2745	12.80916638	6.19				
			Laminaria hyperborea	4.202499906	6.035811	12.74162	15.57166632	9.389				
40	Phytoplankton	-	-	-	-	-	-	-	12.5	6	6	-
